# Nomenclatural and taxonomic updates in Rourea subgen. Rourea sect. Multifoliolatae (Connaraceae)

**DOI:** 10.3897/phytokeys.169.54297

**Published:** 2020-12-08

**Authors:** Cassio A. P. Toledo, Vinicius Castro Souza, Eve J. Lucas

**Affiliations:** 1 Programa de Pós-Graduação em Biologia Vegetal. Instituto de Biologia, Universidade Estadual de Campinas-UNICAMP, Rua Monteiro Lobato, 255, Campinas, SP. CEP: 13083-862, Brazil Universidade Estadual de Campinas-UNICAMP Campinas Brazil; 2 Departamento de Ciências Biológicas. Escola Superior de Agricultura “Luiz de Queiroz”-ESALQ. Universidade de São Paulo-USP, Av. Pádua Dias, 11, Piracicaba, SP. CEP: 13428-900, Brazil Universidade de São Paulo-USP Piracicaba Brazil; 3 Royal Botanic Gardens, Kew, Richmond, Surrey TW9 3DS, UK Royal Botanic Gardens Richmond United Kingdom

**Keywords:** Brazil, Cnestideae, new species, Oxalidales, systematics, typification

## Abstract

The pantropical genus *Rourea* Aubl. (Connaraceae) is composed of ca. 70 species, most of which occur in the Neotropics. *Rourea* is currently subdivided into three subgenera, with the American taxa included in Rourea subgen. Rourea. Forero (1976) recognised six sections for the species of the New World, with Rourea subgen. R. sect. Multifoliolatae being exclusive to Brazil, characterised by multifoliolate leaves, relatively small leaflets and the staminal tube (0.8–)1–1.5 mm long. Following Forero’s (1976) treatment, additional botanical collections have become available in Brazilian herbaria, allowing re-evaluation of species concepts. This work recognises and revises 12 species in this section, mainly restricted to southeastern Brazil and southern Bahia. A nomenclatural and taxonomic study of these species is here presented, including an identification key, morphological descriptions, illustrations and geographic distribution maps. A new species is also described.

## Introduction

*Rourea* Aubl. is the second largest genus in Connaraceae and includes about 70 species (Lemmens 1989a), mainly from lowland tropical forests and savannahs of South America, Central Africa and Asia (Leenhouts 1958; Jongkind 1989; Lemmens 1989a). Its centre of species richness lies on the Neotropics, especially in the Amazon and Atlantic domains, comprising ca. 45 American species (Forero 1983). *Rourea* differs morphologically from other Connaraceae genera by the leaves usually 5 or more foliolate, petals epunctate, flowers 5-carpelate, fruits with calyx accrescent and seeds without endosperm (Forero 1983; Lemmens et al. 2004).

The most recent phylogenetic study and currently accepted classification of Connaraceae was an early cladistic study, based exclusively on morphological evidences (Lemmens 1989b). Four tribes were recognised in the family, with *Rourea* included in Cnestideae Planch., sister to a clade formed by the genera *Cnestidium* Planch. and *Cnestis* Juss.

The circumscription of *Rourea* has varied greatly since the publication of the genus by Aublet (1775). De Candolle (1825), for example, treated the genus as a synonym of *Connarus* L. Later, Planchon (1850) included *Rourea* within the tribe Connareae and described new genera and recognised others that are currently treated in synonymy of *Rourea*, such as *Bernardinia* Planch., *Byrsocarpus* Schumach. and *Roureopsis* Planch. Schellenberg (1938) was even more liberal: he followed Planchon’s concepts and segregated additional taxa from *Rourea*, which resulted in the description of new generic names (e.g. *Santaloides* G. Schellenb). On the other hand, Baillon (1869) and Leenhouts (1958) adopted a broader concept for the genus and placed several genera – including some mentioned above – in *Rourea*, resulting in new synonymisations and combinations. Most of their contributions – although with minor changes – have been followed in modern taxonomic treatments (Forero 1983; Jongkind 1989; Lemmens et al. 2004).

Infrageneric classification of *Rourea* has also varied depending on author’s concept. Planchon (1850) subdivided the genus into two sections, Rourea sect. Dalbergioideae and R. sect. Mimosoideae, with the former represented by the American species and the latter, African and Asian species. Leenhouts (1958) proposed another classification for the genus and recognized three subgenera: Rourea subgen. Rourea, R. subgen. Jaundea (Gilg) Leenh. and R. subgen. Palliathus Leenh. In this subdivision, the American species were included in the former, together with some taxa from western Africa. Forero (1976) adopted Leenhouts’s classification and, while preparing a revision of *Rourea* to the New World, divided the American species into six sections (Rourea subgen. Rourea sect. Rourea, R. subgen. R. sect. Adenophorae G. Schellenb., R. subgen. R. sect. Cordatae Forero, R. subgen. R. sect. Glabrae G. Schellenb., R. subgen. R. sect. Indutae G. Schellenb. and R. subgen. R. sect. Multifoliolatae Forero). Morphological distinction of these sections was mainly based on number of leaflets, leaflet and calyx indumentum and length of the staminal tube.

This treatment includes the species belonging to Rourea subgen. Rourea sect. Multifoliolatae, which are restricted to Brazil and morphologically characterised by the leaves (3–)9–41-foliolate, leaflets usually oblong or narrowly elliptic and smaller than those found in other neotropical *Rourea* and flowers with staminal tubes (0.8–)1–1.5 mm long (Forero 1976, 1983 – with some modifications).

After Forero’s (1976) revisional work, new collections of R. subgen. Rourea sect. Multifoliolatae have been available in Brazilian herbaria thanks to modern field expeditions in the country. This has allowed identification of overlooked morphological characters and, thus, re-evaluation of species concepts in this group. It is also of note that many species of this section are very common in the Brazilian territory, although widely misidentified in herbarium collections. This study presents morphological descriptions of the referred taxa, an identification key, illustrations, geographic distribution maps and detailed taxonomic and nomenclatural notes, along with a new species.

## Methods

The present study was primarily developed based on consultation of literature specific to the genus (Planchon 1850; Baker 1871; Schellenberg 1938; and Forero 1976, 1983) and analysis of specimens from the following herbaria (acronyms according to Thiers 2020): ALCB, BHCB, BR, C, CEN, CEPEC, COL, CVRD, EAC, ESA, F, G, HUEFS, HUEG, HUFU, HUTO, IAN, K, M, MBM, MBML, MG, MO, MPU, NY, P, R, RB, S, SPF, UB, UEC, US, VIC, VIES and W. Fieldwork was carried out by the first author, which allowed collection of botanical samples, photographs and observation of species in their natural habitat. These expeditions took place in Brazil, comprising the municipalities of Conceição da Barra and Guarapari (Espírito Santo) and the district of Gama (Distrito Federal).

General morphological terms mainly follow Font Quer (1953) and Radford et al. (1974), while the venation pattern is based on Ellis et al. (2009) and inflorescence architecture on Weberling (1992).

Geographic distribution maps were prepared using ArcGIS 10.5 (ESRI 2016), based on the localities indicated on herbarium sheet labels. The lists of specimens examined follow alphabetical order by states, then by collectors.

While referring to Brazilian states, the following abbreviations were adopted: Bahia (BA), Distrito Federal (DF), Espírito Santo (ES), Minas Gerais (MG) and Rio de Janeiro (RJ).

## Taxonomic treatment

### Rourea Aubl. subgen. Rourea sect. Multifoliolatae

Taxon classificationPlantaeOxalidalesConnaraceae

Forero, Mem. New York Bot. Gard. 26(1): 37. 1976.

883E1D54-0B44-5475-9FE2-1584E871CAEC

#### Type.

*Rourea blanchetiana* (Progel) Kuhlm.

#### Description.

***Lianas***, subshrubs, shrubs or scandent shrubs, rarely treelets, (0.35–) 0.5–4(–7) m tall; branchlets usually lenticelate. ***Leaves*** alternate, (3–)9–41-foliolate, loosely disposed or congested, without stipules; petiole and rachis with glandular trichomes or eglandular; ***leaflets*** subsessile or pulvinulus 1–2 mm long; blades usually oblong, narrowly elliptic or narrowly ovate, less frequently elliptic, ovate or narrowly obovate, occasionally orbicular on basal leaflets, chartaceous, less frequently coriaceous or membranaceous, abaxially brownish, greenish or glaucous, rarely vinaceous, glabrous, subglabrous, hirsute or villous, base slightly asymmetric to asymmetric, rarely symmetric, apex usually rounded or narrowly rounded, less frequently obtuse, rarely acute (apical leaflets), margin flat, slightly revolute or revolute; secondary venation brochidodromous, tertiary venation reticulate. ***Inflorescences*** in cymes, occasionally panicles or thyrses, axillary or subterminal, rarely terminal; peduncle, rachis and lateral branches (if present) with glandular trichomes or eglandular. ***Flowers*** loosely disposed or congested apically; pedicel with glandular trichomes or eglandular; sepals 5, slightly connate at base, outer surface with glandular trichomes or eglandular; petals 5, epunctate; stamens 10, connate at base by (0.8–)1–1.5 mm, shorter series 5, epipetalous, longer series 5, episepalous, anthers rimose; carpels 5, only 1 developing into a fruit, free, sessile, stigma lobate, ovules 2, only 1 developing into a seed. ***Fruits*** follicular, ellipsoid, straight, calyx accrescent, sepals ascending; ***seeds*** 1, ellipsoid, black, without endosperm, arillode covering the base.

#### Diversity and distribution.

Rourea subgen. R. sect. Multifoliolatae is composed of 12 species exclusively found in the Brazilian states of Bahia, Distrito Federal, Espírito Santo, Minas Gerais and Rio de Janeiro.

#### Recognition.

**Figure 1.**
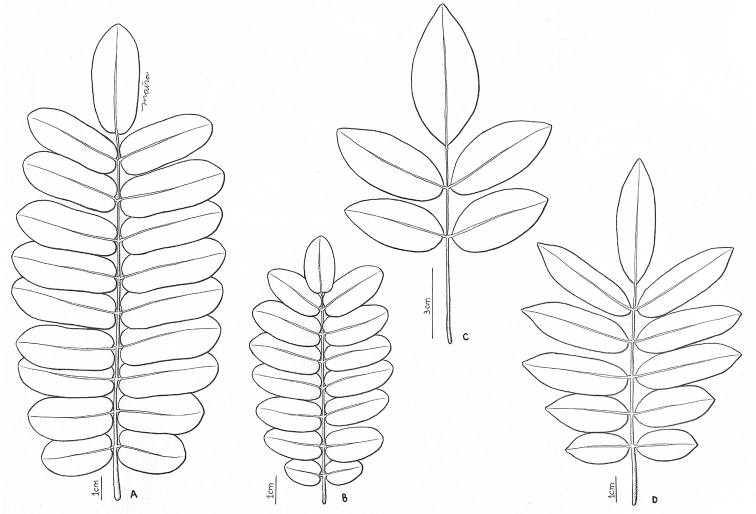
Leaves in Rourea subgen. R. sect. Multifoliolatae: **A***R. discolor***B***R. martiana***C***R. tenuis***D***R. cnestidifolia*.

This section is characterised by multifoliolate leaves, leaflets relatively small, normally oblong or narrowly elliptic with rounded apex (Fig. 1), inflorescences mostly with glandular trichomes and stamens connate at base by (0.8–)1–1.5 mm. Species of this section vegetatively resemble several Leguminosae due to the characteristics cited above, but differ because Connaraceae members do not bear stipules.

### Key to the species of Rourea subgen. Rourea sect. Multifoliolatae

**Table:** 

1	Plants eglandular	**2**
–	Plants with glandular trichomes on inflorescence rachis, pedicel and/or outer surface of sepals	**5**
2	Leaves 3–7(–9)-foliolate; calyx covering two thirds of the fruit	***R. macrocalyx***
–	Leaves 7–41-foliolate; calyx covering one third of the fruit	**3**
3	Inflorescence rachis 3.5–11.5 cm long	***R. discolor***
–	Inflorescence rachis up to 1.8 cm long	**4**
4	Leaves 11–17(–41)-foliolate; inflorescence rachis sparsely pubescent to pubescent; ovary hirsute	***R. bahiensis***
–	Leaves 7–11-foliolate; inflorescence rachis glabrous or subglabrous; ovary hirsute only on one side	***R. barbata***
5	Leaves 27–39-foliolate; leaflets abaxially glaucous; petals 9–14 mm long	***R. blanchetiana***
–	Leaves 3–27-foliolate; leaflets abaxially greenish or brownish; petals (4–)5–8(–9) mm long	**6**
6	Subshrubs	**7**
–	Lianas, shrubs or scandent shrubs	**8**
7	Subshrubs erect; leaflets coriaceous; inflorescence rachis 4–9.5 cm long; fruits 1–1.4 × 0.4–0.6 cm, completely velutinous externally	***R. chrysomalla***
–	Subshrubs prostrate; leaflets chartaceous; inflorescence rachis 1.3–2.2 cm long; fruits 0.8–1×0.3–0.4 cm, sparsely hirsute externally	***R. prostrata***
8	Leaves 5–7-foliolate	**9**
–	Leaves 9–27-foliolate	**10**
9	Petiole 1.3–2.2 cm long; basal pair of leaflets 1.4–2 cm long; inflorescences in cymes	***R. diamantina***
–	Petiole 2.7–7 cm long; basal pair of leaflets 2.3–7.7 cm long; inflorescences in thyrses or panicles	***R. tenuis***
10	Leaflet apex rounded, rarely obtuse; pedicel ca. 2 mm long	***R. martiana***
–	Leaflet apex obtuse or narrowly rounded, rarely acute or rounded; pedicel 3–14 mm long	**11**
11	Peduncle 2.8–8 cm long; flowers congested apically; central and north-eastern MG	***R. cnestidifolia***
–	Peduncle 0.2–1.7 cm long; flowers loosely disposed; central and southern BA, eastern ES and eastern and central RJ	**12**
12	Branchlets subglabrous or sparsely pubescent; leaves 5–9(–13)-foliolate; rachis 3–6(–8.5) cm long; petals 2.5–3 mm wide; central BA	***R. diamantina***
–	Branchlets densely velutinous to glabrescent; leaves (9–)15–27-foliolate; rachis 9–24 cm long; petals 1.5–2 mm wide; southern BA, east coast ES and eastern RJ	***R. glazioui***

### Rourea bahiensis

Taxon classificationPlantaeOxalidalesConnaraceae

Forero, Mem. New York Bot. Gard. 26(1): 103. 1976.

CB2009D0-B1C3-57DF-BA7D-5DF8DA25AE30

Fig. 2

#### Type.

**Brazil. Bahia**: Belmonte, mata costeira, 31 Jan 1967 (fr.), *R. P. Belém & R. S. Pinheiro 3225* (***Holotype***: NY barcode NY 00010955!; ***isotypes***: CEPEC!, MG!, NY!, UB!).

#### Description.

***Lianas*** or scandent shrubs, 1–7 m tall; branchlets glabrous or sparsely puberulous, lenticels abundant, conspicuous or inconspicuous. ***Leaves*** (9–)11–17(–41)-foliolate, congested; petiole 0.6–2.2 cm long, villous to glabrescent, eglandular; rachis 3.5–6.5 cm long, villous to glabrescent, eglandular; ***leaflets*** subopposite to alternate, subsessile or pulvinulus ca. 1 mm long; blade of the basal pair of leaflets 0.6–1.1 × 0.5–0.8 cm, orbicular, others 1.1–2.2(–2.4) × 0.7–1 cm, narrowly elliptic, oblong or narrowly ovate, chartaceous or coriaceous, concolorous, rarely discolorous, sparsely villous to glabrescent on both surfaces, more densely on midvein, abaxially brownish or greenish, adaxially dull, base slightly asymmetric, cordate, subcordate or rounded, apex rounded, margin flat to revolute, glabrous or ciliate; midvein abaxially slightly prominent, adaxially flat, secondary veins 4–5 pairs, flat on both surfaces, tertiary veins flat on both surfaces. ***Inflorescences*** in axillary or ramiflorous cymes or determinate thyrses; bracts ca. 2 mm long; peduncle 1–10 mm long, sparsely pubescent to pubescent, eglandular; rachis 0.3–1.7 cm long, sparsely pubescent to pubescent, eglandular; lateral branches 0.8–2.5 cm long, glabrous or pubescent, eglandular. ***Flowers*** loosely disposed; buds 2–3 × 2 mm, ellipsoid or obovate; pedicel 7–12 mm long, eglandular, 1–2 bracteoles located up to the lower half, persistent; sepals 4–4.5 × 1.5 mm, chartaceous, ovate, outer surface glabrous, subglabrous or sparsely pubescent, eglandular, inner surface glabrous or subglabrous, margin ciliate, more densely at the apex; petals 5–6 × 1.5–2 mm, narrowly elliptic or narrowly obovate, glabrous on both surfaces; stamens connate at base by 0.8–1 mm, shorter series 2–4 mm long, longer series 2.5–6 mm long, glabrous; ovary 1–1.2 mm long, hirsute, style 2–2.5 mm long, sparsely hirsute, glabrous only at the apex, stigma peltate, bilobate. ***Fruits*** 1.1–1.3 × 0.4–0.6 cm, yellowish or reddish, outer surface sparsely puberulous, inner surface glabrous, apex obtuse, style partially persistent, calyx covering one third of the fruit; ***seeds*** ca. 0.8–1 × 0.3–0.5 cm, arillode orangish.

**Figure 2.**
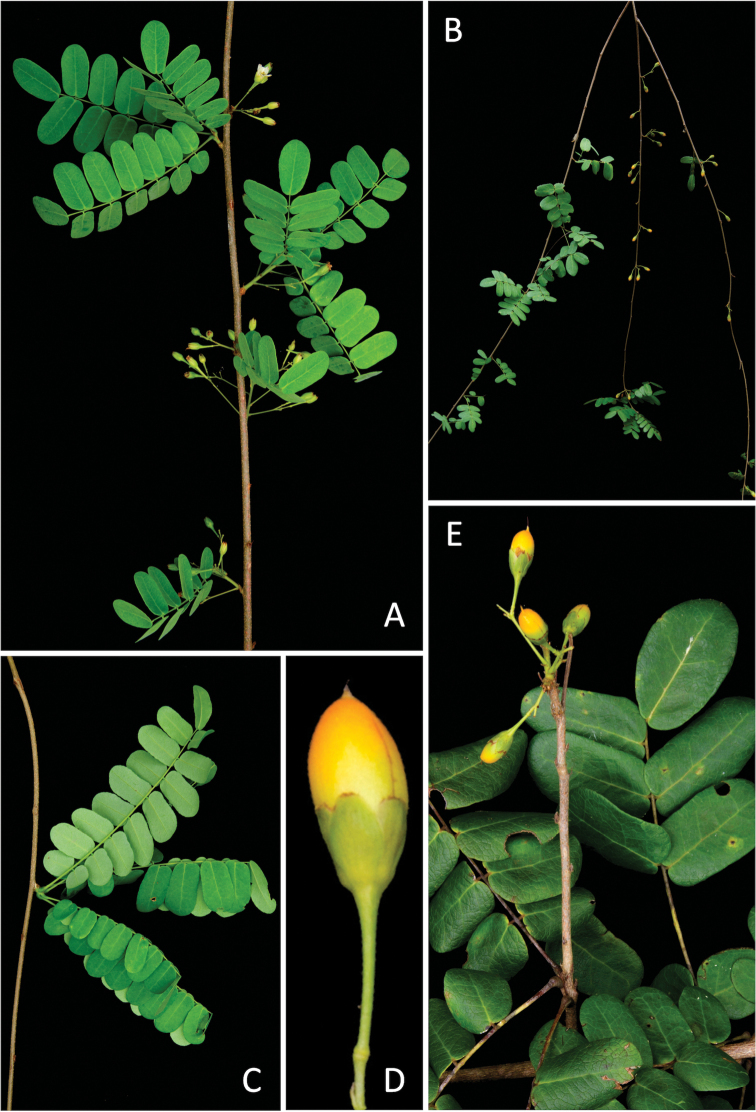
*Rourea bahiensis*: **A** flowering branchlet **B** habit **C** congested leaves **D** fruit **E** fruiting branchlet.

#### Distribution, habitat and phenology.

**Figure 3.**
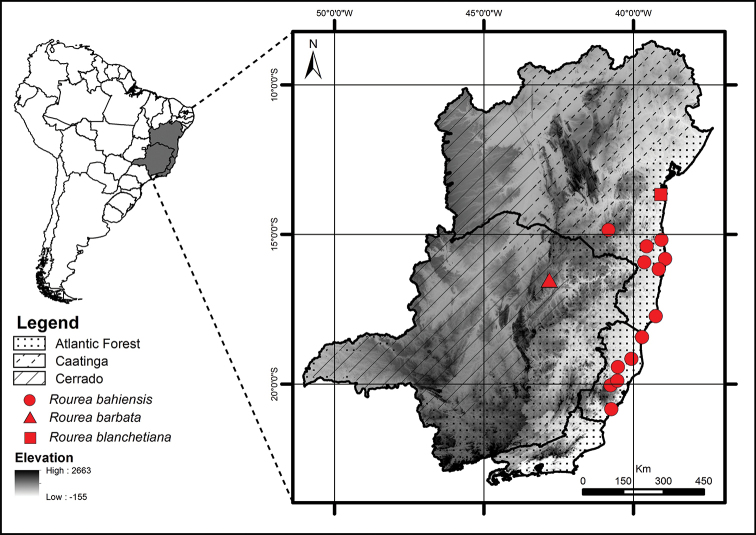
Geographic distribution of *Rourea bahiensis* (circles), *R. barbata* (triangles) and *R. blanchetiana* (squares).

This species has only been found in Bahia and Espírito Santo (Fig. 3). In BA, populations of *R. bahiensis* are distributed in the southern portion of the state, occurring mainly in coastal areas from the municipality of Una to Porto Seguro and Alcobaça. In ES, specimens have been collected both in the interior of the state and coastal zones, ranging from northern to southern portions. Individuals of *Rourea bahiensis* are lianas or scandent shrubs up to 7 m tall. This species occurs in the Atlantic Rain Forest, growing on “Tabuleiro” or swamp forests, although sometimes it is also found in degraded areas. Specimens have been collected with flowers from October to November and with fruits from September to January.

#### Specimens examined.

**Brazil. Bahia**: Porto Seguro, RPPN Veracruz (Veracel Celulose), rodovia que atravessa a reserva, antiga entrada para Santa Cruz Cabrália, ca. 2 km do centro de visitantes, 16°09'27"S, 39°09'15"W, 02 Nov 2001 (fl.), *J. G. Jardim & R. V. Lopes 3952* (CEPEC, HUEFS, NY); Itapebi, estrada para o distrito de Caubi, ca. 7 km da BR 101, Fazenda Palmeiras, ca. 10.5 km na entrada, 15°55'36"S, 39°38'09"W, 04 Nov 2001 (fl.), *J. G. Jardim & R. V. Lopes 3954* (HUEFS, NY); Una, Reserva Biológica de Una (REBIO de Una), entrada ca. 45 km S de Ilhéus, margem da estrada que leva à sede da reserva, 15°10'46"S, 39°03'50"W, 13 Jan 2001 (fr.), *F. Juchum & E. Forero 116* (CEPEC, NY, RB); Vitória da Conquista, Reserva do Poço Escuro, 26 Nov 2011 (fl.), *L. C. Marinho et al. 218* (HUEFS); Camacan, trilha da Bapeba, 15°23'30"S, 39°33'35"W, 02 Apr 2009 (fr.), *R. de O. Perdiz et al. 357* (CEPEC); Caravelas, rodovia BR 418, a 27 km SW de Alcobaça, 16 Sep 1978 (fr.), *T. S. dos Santos et al. 3361* (CEPEC). **Espírito Santo**: Marilândia, Liberdade (Água Viva, Pedra do Cruzeiro), prop.: Aguilar A. Lovucini, 18 Jan 2006 (fl., fr.), *V. Demuner et al. 1626* (MBML, UB); Conceição da Barra, próximo à Itaúnas, 18°25'41"S, 39°42'56"W, alt. 10 m, 30 Oct 2014 (fr.), *J. E. Q. Faria & T. N. C. Vasconcelos 4231* (UB); Linhares, Reserva Natural da Companhia Vale do Rio Doce, Aceiro Aracruz, primeira elevação após o entroncamento das 3 reservas, CVRD, Sooretama e Aracruz-Fazenda Calliman, 31 Oct 2007 (st.), *F. L. R. Filardi 776* (RB); Reserva Natural Vale, estrada Farinha Seca, RFL-001/80, Bloco E, Trat 1, 30 Oct 2010 (fl.), *D. A. Folli 5407* (CVRD, ESA); Estrada Gávea, 30 Oct 2003 (fl.), *D. A. Folli 4659* (CVRD, ESA); Piuma, Apr 1993 (fr.), *Helder José s. n.* (SPF 77169); Santa Maria de Jetiba, Rio nove, Sítio de L. Kollmann, 26 Jan 2004 (fl.), *L. Kollmann 6361* (BHCB, MBML, UB); Santa Teresa, Reserva Biológica Augusto Ruschi, 05 Dec 2002 (fl.), *L. Kollmann & E. Bausen 5823* (MBML); Santa Teresa, Nova Lombardia, Reserva Biológica Augusto Ruschi. Altitude 800 m, 28 Nov 2001 (fl.), *L. Kollmann et al. 5072* (MBML); Itaúnas, área da Fíbria com plantação de eucalipto, 18°29'27"S, 39°44'12"W, 21 Oct 2018 (fl., fr.), *C. A. P. Toledo & N. C. Bígio 399* (ESA); Estrada para Lombardia, 25 Apr 2002 (fr.), *R. R. Vervloet & E. Bausen 194* (MBML, UB); Trilha antiga, sede lado direiro, 26 Mar 2003 (fr.), *R. R. Vervloet & E. Bausen 2053* (MBML, UB).

#### Recognition and notes.

*Rourea bahiensis* is morphologically similar to *R. prostrata* due to their narrowly elliptic or oblong leaflets usually measuring 0.9–2.2 × 0.5–1 cm. However, they are differentiated because *R. bahiensis* is a lianescent species without glandular trichomes, while *R. prostrata* is a prostrate subshrub with glandular trichomes. *Rourea bahiensis* can also be mistaken for *R. discolor*, from which it mainly differs by the smaller leaflets (1.1–2.2(–2.4) × 0.7–1 cm) and inflorescence rachis 0.3–1.7(–3.7) cm long vs. larger leaflets ((1.3–)2.2–4.5(–5.8) × (0.8–)1.2 × 1.7(–2.3) cm) and inflorescence rachis 3.5–11.8 cm long in *R. discolor*. Additionally, the petiole and leaf rachis are subglabrous to villous and the fruits are smaller (1.1–1.3 × 0.4–0.5 cm) in *R. bahiensis*, while, in *R. discolor*, the petiole and leaf rachis are glabrous and the fruits are larger (1.2–1.7 × 0.4–0.6 cm).

This species varies considerably in the characteristics of leaflet size, indumentum and texture. Some specimens, for example, have larger and chartaceous leaflets, which are usually sparsely villous abaxially; the opposite of this combination is composed of specimens with smaller, coriaceous and glabrous leaflets. This seems a common variation in the species probably related to branch and leaf development as few materials were found with the two conditions mentioned above (e.g. *J. G. Jardim et al.* 3952). Additionally, deciduousness of trichomes may explain why some leaf blades are glabrous and others, sparsely villous.

### Rourea barbata

Taxon classificationPlantaeOxalidalesConnaraceae

C. Toledo, Phytotaxa 408(2): 118. 2019.

77C790C5-44ED-563B-AF56-1738F55EDC65

Illustration: Forero (2003 ) and Toledo and Souza (2019)

#### Type.

**Brazil. Minas Gerais**: Grão Mogol, cerrado na estrada para o rio Ventania, ca. 16°32'S–42°49'W, ca. 900 m alt., 5 Nov 1990 (fl.), *J. R. Pirani CFCR13358* (***Holotype***: SPF barcode SPF 69503!; ***isotypes***: ESA!, NY!).

#### Description.

***Shrubs***, ca. 1.5 m tall; branchlets glabrous or subglabrous, lenticels absent. ***Leaves*** 7–11-foliolate, congested; petiole 0.8–1.7 cm long, glabrous or subglabrous, eglandular; rachis 3.5–4.4 cm long, glabrous or subglabrous, eglandular; ***leaflets*** opposite to subopposite, subsessile; blade of the basal pair of leaflets 1–1.6 × 0.6–0.9 cm, ovate, others 1.3–3.2 × 0.8–1.4 cm, narrowly ovate or narrowly elliptic, chartaceous, discolorous, glabrous on both surfaces, abaxially vinaceous, adaxially greyish, slightly shining, base symmetric or slightly asymmetric, subcordate, apex obtuse, rounded or narrowly rounded, margin slightly revolute, glabrous; midvein abaxially prominent, adaxially slightly impressed, secondary veins 5–7 pairs, slightly prominent on both surfaces, tertiary veins slightly prominent on both surfaces. ***Inflorescences*** in axillary or pseudoterminal cymes; bracts 2–3 mm long; peduncle 0.2–3.5 cm long, glabrous or subglabrous, eglandular; rachis 0.7–1.8 cm long, glabrous or subglabrous, eglandular. ***Flowers*** loosely disposed or congested apically; buds 4–3 × 3 mm, orbicular; pedicel 6–12 mm long, eglandular, 2 bracteoles located up to the lower half, persistent; sepals 4–4.5 × 2 mm, chartaceous, ovate or narrowly ovate, outer surface subglabrous, eglandular, inner surface glabrous or subglabrous, margin ciliate, more densely at the apex; petals 5.5–6 × 2 mm, oblong or narrowly obovate, glabrous on both surfaces; stamens connate at base by ca. 1 mm, shorter series ca. 3.5 mm long, longer series 5–5.5 mm long, glabrous; ovary 1–1.2 mm long, densely hirsute only on one side, glabrous or almost so elsewhere, style ca. 2 mm long, sparsely hirsute at base, glabrescent towards the apex, stigma peltate, bilobate. ***Fruits*** unknown.

#### Distribution, habitat and phenology.

This species is only known from the type location (Fig. 3). It has a shrubby habit and grows in areas of cerrado, at ca. 900 m altitude. The only flowering specimen was collected in November.

#### Recognition and notes.

*Rourea barbata* is mainly recognised by the glabrous leaflets and the ovary, which is hairy only on one side of the structure. It is similar to *R. martiana* due to the number, size and shape of leaflets, but these are glabrous in *R. barbata* and hirsute or villous in the latter.

Forero (2003) identified the type of *R. barbata* as *R. discolor*; however, the former differs by the leaves 7–11-foliolate, inflorescence rachis 0.7–1.8 cm long and the ovary with a tuft of trichomes, while the latter has leaves 9–29-foliolate, inflorescence rachis 3.5–11.5 long and ovary completely hirsute.

### Rourea blanchetiana

Taxon classificationPlantaeOxalidalesConnaraceae

(Progel) Kuhlm., Arq. Inst. Biol. Veg. 1: 40. 1934.

8945B6BC-220A-5B83-B4F8-5D558F3F43D8

Illustration: Progel (1877) and Schellenberg (1938)

Eichleria blanchetiana Progel, in Martius, Fl. Bras. 12(2): 518. 1877.

#### Type.

**Brazil. Bahia**: S. d. (fl., fr.), *J. S. Blanchet 1050* (***Lectotype***: BR barcode BR 00000528168!, designated here; ***isolectotypes***: G!, S!, W!).

#### Description.

***Lianas***: branchlets sparsely hirsute to hirsute, lenticels abundant, inconspicuous. ***Leaves*** 27–39-foliolate, loosely disposed; petiole 1.4–2.2 cm long, sparsely hirsute to hirsute, with glandular trichomes; rachis (8–)10–14.5 cm long, sparsely hirsute to hirsute, with glandular trichomes; ***leaflets*** subopposite to alternate, pulvinulus ca. 1 mm long; blade of the basal pair of leaflets 0.9–1.5 × 0.6–0.9 cm, orbicular or oblong, others 1.3–2.9 × 0.6–1 cm, oblong, the apical ones elliptic, membranaceous to subchartaceous, discolorous, abaxially glabrous, subglabrous or sparsely hirsute on the midvein, glaucous, adaxially glabrous, dull, base symmetric or slightly asymmetric, rounded or subcordate, obtuse in the apical leaflet, apex rounded, rarely truncate, obtuse in the apical leaflet, margin flat, glabrous; midvein abaxially slightly prominent, rarely prominent, adaxially slightly impressed, rarely impressed, secondary veins 5–7 pairs, abaxially slightly prominent or flat, adaxially flat, tertiary veins flat on both surfaces. ***Inflorescences*** in axillary cymes; bracts ca. 1 mm long; peduncle (2–) 3.5–6.7 cm long, densely hirsute, with glandular trichomes; rachis 0.5–2.2 cm long, densely hirsute, with glandular trichomes. ***Flowers*** congested apically; buds 3–6 × 2–3 mm, elliptic; pedicel 8–13 mm long, with glandular trichomes, 1–2 bracteoles located in the middle portion, deciduous; sepals 4–6 × 1–1.5 mm, chartaceous, lanceolate, outer surface hirsute, with glandular trichomes, inner surface subglabrous, margin ciliate; petals 9–14 × 2 mm, oblong or narrowly obovate, glabrous on both surfaces; stamens connate at base by ca. 1 mm, shorter series ca. 1 mm long, longer series ca. 2 mm long, with sparse glandular trichomes; ovary ca. 1.2 mm long, hirsute, style ca. 2.5 mm long, sparsely hirsute, subglabrous or glabrous only at the apex, stigma peltate, bilobate. ***Fruits*** 1.2 × 0.6–0.7 cm, colour not seen, outer surface sparsely hirsute, inner surface not seen, apex acuminate, style deciduous, calyx covering one third of the fruit; ***seeds*** ca. 1 × 0.4 cm, arillode colour not seen.

#### Distribution, habitat and phenology.

Most of the known specimens of *R. blanchetiana* are old collections with no precise locations. The most recently analysed specimen was collected in the municipality of Nilo Peçanha, which is located in southern Bahia (Fig. 3). Therefore, it seems that *R. blanchetiana* is a rare species and most likely restricted to the south portion of Bahia. This is a lianescent species growing on ombrophilous forests of the Atlantic domain. The specimen *J. S. Blanchet* s. n. (NY barcode NY00393553) was collected with flowers and fruits in August.

#### Specimens examined.

**Brazil. Bahia**: Locality unknown: Aug 1835 (fl., fr.), *J. S. Blanchet s. n.* (NY barcode NY00393553); s. d. (fl.), *J. S. Blanchet s. n.* (?) (P barcode P05487855). Nilo Peçanha, estrada a ca. 4 km da comunidade Quilombola de Jatimane na estrada para Ituberá, fazenda Outeiro do Chapeú, 13°40'06"S, 39°04'50"W, 14 Apr 2012 (st.), *L. P. de Queiroz & F. H. F. Nascimento 15452* (HUEFS); Locality unknown, s. d. (fl.), *J. S. Blanchet 297* (P barcode P05487854); s. d. (fl.), *P. Salzmann s. n.* (MO 1704495, P barcode P05487856).

#### Recognition and notes.

*Rourea blanchetiana* is easily recognised by the leaves with 27–39 leaflets, which are usually membranaceous, oblong and abaxially glaucous, aside from the flowers congested in the inflorescences, comparatively larger peduncle and petals 9–14 mm long. It is similar to *R. discolor* due to the leaflets abaxially glaucous and pedicel relatively long, but differs by the hirsute inflorescence rachis, elliptic flower buds and petals 9–14 mm long vs. glabrous or subglabrous inflorescence rachis, orbicular or ovate flower buds and petals (4–)6–8 mm long.

In the protologue of the basionym of *Rourea blanchetiana* from *Flora Brasiliensis*, the only specimen cited by Progel (1877) was Blanchet 1050, without indication of herbarium. Schellenberg (1938) inadvertently selected the lectotype from P. However, of the six specimens of *R. blanchetiana*, collected by Blanchet in P, none is annotated with number 1050. Specimens of Blanchet 1050 were found in BR, G, S and W and only the collection from G is fruiting, the rest, only flowering. Of these, the specimen from BR has an original label of “Herbarium Martii”, identified as “*Eichleria blanchetiana* Prog. in Mart. fl. Br.” and bears an attached pencil drawing containing floral details of the referred species. The characteristics of the branchlet of this specimen (number and shape of leaflets, inflorescence architecture and flower structures) and the detailed drawing match accurately with the illustration in Progel (1877, plate 116). In addition, considering that Progel worked in M and the original *Flora Brasiliensis* collection from M was sold to BR, then the specimen Blanchet 1050, deposited in the latter, seems the best candidate to be lectotype of *R. blanchetiana*, which is here proposed.

*Rourea blanchetiana* was originally described in Oxalidaceae (Progel 1877). The author published the basionym under his new genus *Eichleria*, based on the similarity between Connaraceae and Oxalidaceae with respect to the imparipinate leaves and heterostylous flowers with apocarpous gynoecium. Kuhlmann (1934) firstly noticed this confusion and proposed the prompt combination after concluding that the species bear two ovules with marginal placentation, characteristic of Connaraceae. The close relationship between Connaraceae and Oxalidaceae has been confirmed by modern phylogenetic analyses and they form a monophyletic group within Oxalidales (APG IV 2016); Connaraceae differs morphologically from Oxalidaceae by the completely free carpels and follicular fruits.

### Rourea chrysomalla

Taxon classificationPlantaeOxalidalesConnaraceae

Glaz. ex G. Schellenb., in Engler, Pflanzenreich IV. 127(Heft 103): 196. 1938.

338B82AA-363E-53C2-8F11-C9C8083A90FA

Fig. 4

#### Type.

**Brazil. Goiás**: Chemin du Rio Paranauá á Chico Lobo, dans les campos, 08 Nov 1894 (fr.), *A. F. M. Glaziou 20871* (***Lectotype***: P barcode P02274085!, designated here; ***isolectotypes***: F!-frag., G, K!, MPU!, P!, R!, RB!, S!).

#### Description.

***Subshrubs*** erect, 0.35–0.65(–0.8) m tall; branchlets densely velutinous, lenticels absent. ***Leaves*** 9–17-foliolate, loosely disposed; petiole 0.5–2 cm long, velutinous, with glandular trichomes; rachis 4.5–10 cm long, velutinous, with glandular trichomes; ***leaflets*** opposite to subopposite, sessile; blade of the basal pair of leaflets (0.9–)1.2–1.8 × (0.4–)0.8–1.6 cm, orbicular or ovate, others 1.6–3.7 × 0.9–1.6 cm, ovate, narrowly elliptic or narrowly ovate, the apical ones usually elliptic, coriaceous, discolorous, abaxially hirsute to densely hirsute, brownish or greenish, adaxially sparsely hirsute to hirsute, dull, base symmetric or slightly asymmetric, cordate, subcordate or rounded, apex obtuse or rounded, margin flat or slightly revolute, ciliate; midvein abaxially prominent, adaxially flat, secondary veins 4–6 pairs, abaxially prominent or slightly prominent, adaxially flat or slightly prominent, tertiary veins abaxially slightly prominent, adaxially flat or slightly prominent. ***Inflorescences*** in axillary or terminal determinate thyrses or panicles; bracts ca. 3 mm long; peduncle 0.7–3 cm long, densely hirsute, with glandular trichomes; rachis 4–9.5 cm long, densely hirsute, with glandular trichomes; lateral branches 0.7–2(–3) cm long, densely hirsute, with glandular trichomes. ***Flowers*** loosely disposed; buds 4–7 × 3–4 mm, elliptic or broadly elliptic; pedicel 1–4 mm long, with glandular trichomes, 2 bracteoles located up to the lower half, deciduous or persistent; sepals (5–)6–8 × 1.5–2.5 mm, coriaceous, ovate or narrowly elliptic, outer surface densely velutinous or densely hirsute, with glandular trichomes, inner surface subglabrous, margin ciliate; petals 7–9 × 2–3 mm, narrowly elliptic or narrowly obovate, with sparse glandular trichomes on both surfaces; stamens connate at base by ca. 0.8 mm, shorter series 3–5 mm long, longer series 4–6 mm long, with sparse glandular trichomes; ovary 1–1.3 mm long, densely hirsute, style 2–4 mm long, hirsute, stigma peltate, bilobate. ***Fruits*** 1–1.4 × 0.4–0.6 cm, reddish or orangish, outer surface completely velutinous, inner surface subglabrous, apex acuminate, style partially persistent, calyx covering one third or rarely half of the fruit; ***seeds*** 0.7–1 × 0.4 cm, arillode yellowish.

**Figure 4.**
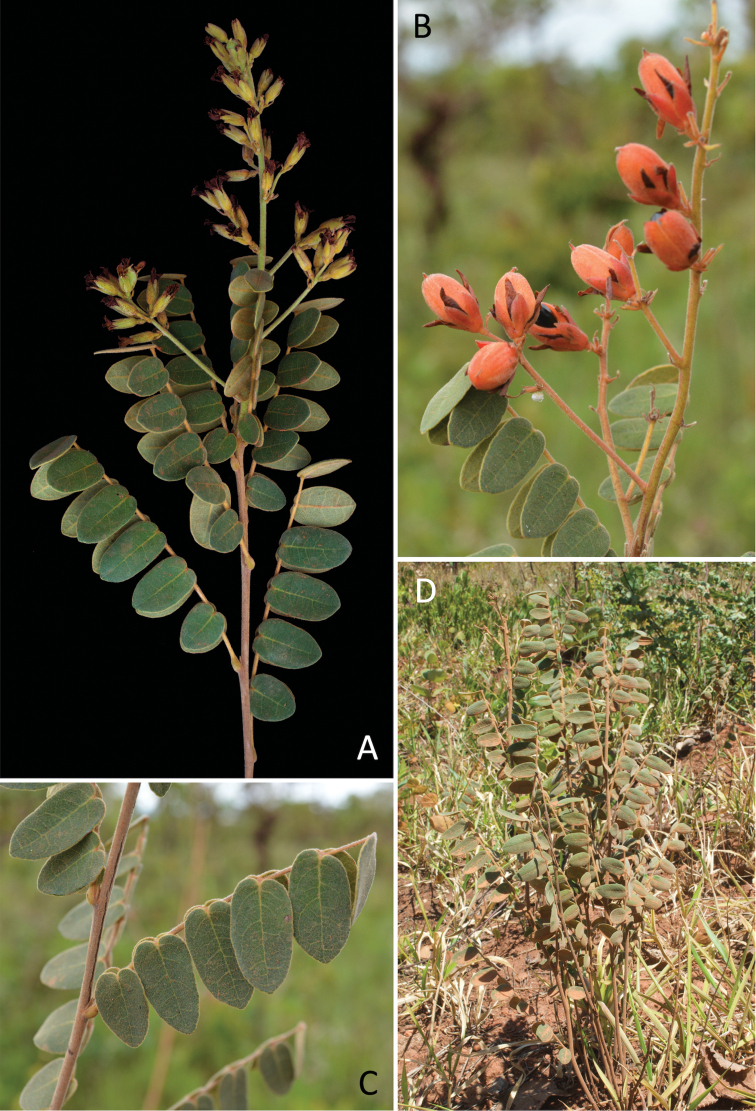
*Rourea chrysomalla*: **A** flowering branchlet **B** fruiting branchlet (photo by Jair Faria) **C** leaves (photo by Jair Faria) **D** habit.

#### Distribution, habitat and phenology.

**Figure 5.**
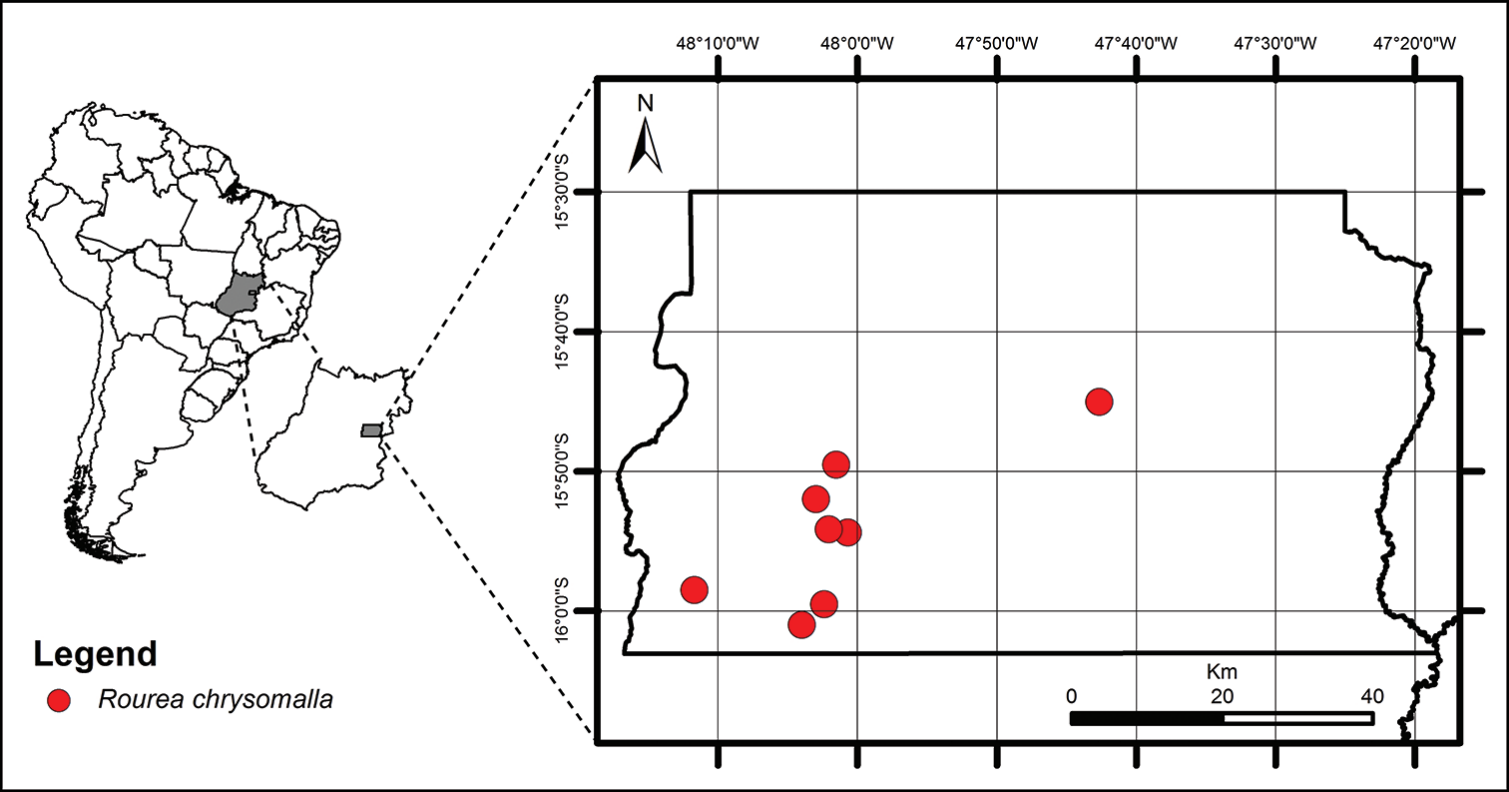
Geographic distribution of *Rourea chrysomalla*.

*Rourea chrysomalla* is apparently restricted to Distrito Federal (DF), in the midwest region of Brazil (Fig. 5). The type specimen label indicates that the material was collected in Goiás state, but it is more likely to have been collected in DF (within the current boundaries of Goiás), which is supported by evidence from several sources. Firstly, the herbarium sheet label of Glaziou’s specimen is from “Chemin du Rio Paranauá, a Chico Lobo”; although the name of this river has been taken as “Parananá”, the penultimate letter more closely resembles a “U”, than an “N”. Secondly, Paranauá stands for Paranoá, a river course from DF, subject of study by Glaziou between 1894 and 1895. This can be confirmed by reports and letters from Glaziou to Missão Cruls (Cruls’ mission), a large scientific expedition in the Brazilian Central Plateau, promoted to investigate the viability of transferring the capital of Brazil to the Midwest (available at: http://doc.brazilia.jor.br/HistDocs/Relatorios/1896-missao-Cruls-Glaziou-lago-Paranoa.shtml). Glaziou sent detailed reports on aspects of vegetation, soil quality and climate around the Paranoá River and clearly demonstrated his advice to dam its riverbed to set up an artificial lake, which was undertaken only in 1959. This lake is called Lago Paranoá and it was probably built following Glaziou’s reports, suggesting that the botanist indeed collected in areas that are today part of DF. It is also of note that Glaziou’s reports are contemporaneous with his collection of *R. chrysomalla*. Lastly, according to the description of his itinerary (Glaziou 1906), the author collected in Goiás from 1894 to 1895 and visited sites around and within what is now DF, indicating that his collections from Goiás also included specimens from DF. This evidence, in addition to the fact that *R. chrysomalla* is only found in DF, suggests that the type was collected in the present DF. Unfortunately, no information on “Chico Lobo” location was found.

In DF, individuals of *R. chrysomalla* can be found in the districts of Brasília, Gama, Riacho Fundo, Sobradinho and Taguatinga. This species is a subshrub up to 65(–80) cm tall, occurring in the central Brazilian Cerrado, more specifically in areas of cerrado *s. s.* or “campo sujo”. *Rourea chrysomalla* is apparently the only species of the genus in which the roots develop a xylopodium. Specimens have been collected with flowers from August to October and in April and with fruits from September to October.

#### Specimens examined.

**Brazil. Distrito Federal**: Sobradinho, 10 Apr 1974 (fl.), *E. P. Heringer 13232* (UB); Taguatinga, Brasília, 15 Aug 1964 (fl.), *E. P. Heringer 9750* (UB, US); Brasília, ca. 15 km E. of Brasília, 30 Oct 1964 (fl.), *H. S. Irwin & T. D. Soderstrom 5724* (NY); Disturbed areas in cerrado, ca. 32 km S.W. of Brasília on road to Anápolis, 04 Sep 1964 (fl.), *H. S. Irwin & T. D. Soderstrom 6001* (NY, P, UB, US); Riacho Fundo, 15 Oct 1999 (fl.), *L. C. Milhomens et al. 03* (UB); Riacho Fundo, (fl.), *L. C. Milhomens et al. 04* (UB); A ca. de 25 km sudoeste de Brasília, caminho para Anápolis, 15 Oct 1999 (fl.), *L. C. Milhomens & W. R. Anderson 05* (HUEG, UB); Coletada em campo sujo de Cerrado, c/ intensa ação antrópica, 15 Oct 1999 (fl.), *L. C. Milhomens 06* (EAC, HUEG, HUFU, HUTO, UB); Riacho Fundo, (st.), *L. C. Milhomens et al. 07* (UB); Coletada em campo sujo de Cerrado, c/ intensa ação antrópica, 15 Oct 1999 (fl.), *L. C. Milhomens 08* (HUTO, UB); CINDACTA, 05 Oct 1999 (fr.), *L. C. Milhomens et al. 09* (UB); CINDACTA, 05 Oct 1999 (fr.), *L. C. Milhomens et al. 10* (UB); CINDACTA, -16.01666705, -48.066667, 05 Oct 1999 (fr.), *L. C. Milhomens et al. 11* (UB); CINDACTA, 05 Oct 1999 (fr.), *L. C. Milhomens et al. 12* (UB); CINDACTA, 05 Oct 1999 (fr.), *L. C. Milhomens et al. 13* (UB); CINDACTA, 05 Oct 1999 (fr.), *L. C. Milhomens et al. 14* (UB); CINDACTA, 05 Oct 1999 (fr.), *L. C. Milhomens et al. 15* (UB); Gama, área pertencente ao CINDACTA, pouco depois do balão de entrada do Gama, 16°01'S, 48°04'W, 05 Oct 1999 (fr.), *L. C. Milhomens et al. 16* (EAC, UB); Fazenda Sucupira, áreas nativas a Oeste da sede do Laboratório-BBGA, 15°52'S, 48°00'W, 08 Nov 1996 (fr.), *R. V. Nunes et al. 59* (CEN); Riacho Fundo, 25 Sep 1998 (st.), *C. E. B. Proença 2063* (UB); Riacho Fundo, (st.), *C. E. B. Proença 2064* (UB); Riacho Fundo, (st.), *C. E. B. Proença 2065* (UB); Riacho Fundo, (fr.), *C. E. B. Proença 2066* (UB); Riacho Fundo, (fr.), *C. E. B. Proença 2067* (UB); Samambaia, Parque Boca da Mata do lado esquerdo da polícia, 15°52'S, 48°03'W, 07 Aug 1995 (fl.), *J. M. de Rezende 49* (CEN); Cerrado senso stricto, estrada de terra na saída da cidade, 15°59'31"S, 48°02'24"W, 10 Oct 2017 (fl.), *C. A. P. Toledo & J. R. L. da Paz 347* (ESA).

#### Recognition and notes.

*Rourea chrysomalla* is easily distinguished by the combination of the following characters: branchlets velutinous, leaflets sessile, coriaceous, abaxially densely hirsute, sepals coriaceous, petals with glandular trichomes and fruits with outer surface completely velutinous. This species can be confused with *R. prostrata* due to their sub-shrubby habit; however, *R. chrysomalla* is an erect subshrub (vs. prostrate subshrub), has coriaceous leaflets (vs. chartaceous), inflorescences usually terminal (vs. axillary) and fruits measuring 1–1.4 × 0.4–0.6 cm, completely velutinous externally (vs. fruits 0.8–1 × 0.3–0.4 cm, sparsely hirsute especially at apex). *Rourea chrysomalla* is also similar to *R. glazioui*, but differs by the subshrubby habit, coriaceous leaflets and petals and stamens with glandular trichomes vs. lianescent habit, chartaceous leaflets and petals and stamens eglandular.

*Rourea chrysomalla* was described by Glaziou (1906, written as “*chrysomala*”), but his work is listed in the Suppressed works (Appendix I) of the *Code* (Turland et al. 2018), so the name is considered not validly published. Schellenberg (1938) validly published *Rourea chrysomalla* and gave credits to Glaziou, so the authorship of the name has been attributed to Glaziou ex Schellenberg. The type (*Glaziou 20871*) indicated by Schellenberg (1938) is from P and, in this herbarium, there are two sheets belonging to this collection. Although each sheet bears the same label from P, only one (barcode P02274085) has an original label with Glaziou’s handwriting indicating location and date, so the two specimens are considered duplicates (Turland et al. 2018, Art. 8.3); then a lectotype is here proposed.

### Rourea cnestidifolia

Taxon classificationPlantaeOxalidalesConnaraceae

G. Schellenb., in Engler, Pflanzenreich IV. 127(Heft 103): 198. 1938.

8627739E-C5A6-5164-B3C3-D53202E7CC4D

Fig. 1D

#### Type.

**Brazil. Minas Gerais**: S. d., *F. Sellow* s. n. (***Holotype***: B†). **Brazil**. **Minas Gerais**: Lagoa Santa, s. d. (fl.), *J. E. B. Warming 1849* (***Lectotype***: K barcode K 000633716!, designated by Forero 1976).

#### Description.

***Shrubs*** or scandent shrubs, 1–2.5 m tall; branchlets hirsute to glabrescent, lenticels abundant, inconspicuous. ***Leaves*** 9–13-foliolate, loosely disposed; petiole 1.9–2.8 cm long, sparsely hirsute to hirsute or sparsely villous, with glandular trichomes; rachis 4–11 cm long, sparsely hirsute to hirsute or sparsely villous, with glandular trichomes; ***leaflets*** opposite to alternate, pulvinulus ca. 1 mm long; blade of the basal pair of leaflets 2.3–4.7 × 1.3–2 cm, ovate, elliptic or oblong, others 4–7.2 × 1.6–3.2 cm, narrowly ovate, oblong or narrowly elliptic, the apical ones always elliptic or narrowly elliptic, chartaceous, slightly discolorous, abaxially hirsute or villous, occasionally subglabrous, more densely in the midvein, brownish or greenish, adaxially glabrous or subglabrous, usually sparsely villous on midvein, slightly shining or dull, base slightly asymmetric to asymmetric, usually symmetric in the apical leaflet, rounded, cordate or subcordate, occasionally acute in the apical leaflet, apex obtuse, acute or narrowly rounded, margin flat, rarely slightly revolute, ciliate or sparsely ciliate; midvein abaxially prominent or slightly prominent, adaxially impressed or slightly impressed, secondary veins 6–7 pairs, abaxially slightly prominent, adaxially flat, tertiary veins abaxially slightly prominent or flat, adaxially flat or slightly prominent. ***Inflorescences*** in axillary cymes, rarely pseudoterminal; bracts 2–3 mm long; peduncle 2.8–8 cm long, hirsute, with glandular trichomes; rachis 0.8–1.9 cm long, hirsute, with glandular trichomes. ***Flowers*** congested apically; buds 2.5–4 × 2–3 mm, orbicular or broadly elliptic; pedicel 3–5 mm long, with glandular trichomes, 2 bracteoles located up to the lower third, deciduous; sepals 4–5 × 2–2.5 mm, chartaceous, ovate, outer surface hirsute, with glandular trichomes, inner surface subglabrous or sparsely sericeous, margin ciliate; petals 7–8 × 2–3 mm, narrowly obovate or narrowly elliptic, glabrous on both surfaces; stamens connate at base by ca. 1.5 mm, shorter series ca. 1.5 mm long, longer series ca. 2.5 mm long, glabrous; ovary 1–1.3 mm long, densely hirsute, style 2–4 mm long, hirsute, stigma peltate, bilobate. ***Fruits*** 1.3–1.4 × 0.4–0.5 cm, orangish, outer surface subglabrous, sparsely hirsute at the apex, inner surface glabrous or subglabrous, apex acuminate, style partially persistent, calyx covering one third of the fruit; ***seeds*** 0.9–1 × 0.4 cm, arillode colour not seen.

#### Distribution, habitat and phenology.

**Figure 6.**
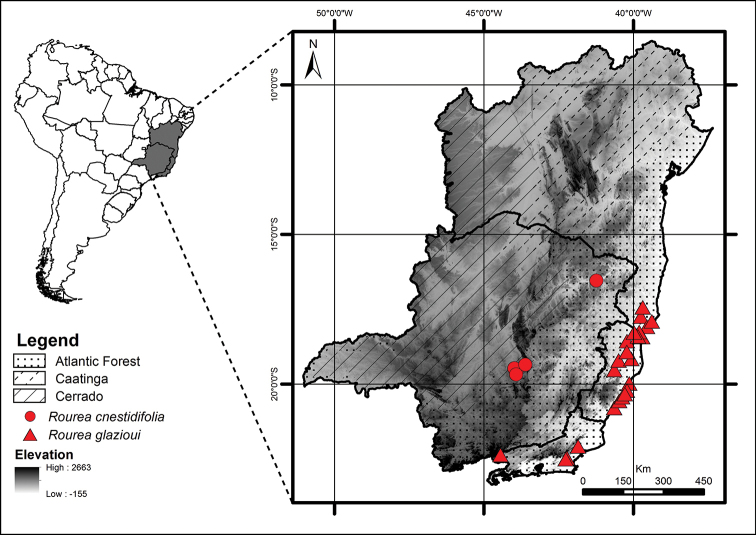
Geographic distribution of *Rourea cnestidifolia* (circles) and *R. glazioui* (triangles).

There are only few records of this species, which seems to be restricted to central and northeastern Minas Gerais (Fig. 6). This shrubby species occurs in the Atlantic Forest or at the transition with the Cerrado, where it is distributed in areas of “Cerradão” and seasonal forests; it grows in rocky or limestone outcrops, at approximately 600–1,000 m altitude. Specimens have been collected with flowers from August to November and with fruits from October to December.

#### Specimens examined.

**Brazil. Minas Gerais**: Rio Doce, localidade Figueira, 11 Sep 1930 (fl.), *J. G. Kuhlmann 348* (IAN, RB); Matozinhos: fazenda Cauaia, 31 Oct 1996 (fr.), *J. A. Lonbardi 1453* (BHCB); Fazenda Castelo da Jagoara, 19°28'12.0"S, 43°58'59.1"W, 683 m alt., 21 Oct 2006 (fr.), *J. C. F. Melo et al. 514* (BHCB, SPF). Vespasiano, região metropolitana de Belo Horizonte, afloramento calcário, adjacente à Lavra da Cia de Cimento Portland Itaú, Dec 1990 (fr.), *M. A. L. Rollo 37* (SPF); Serra do Cipó, Aug 1895 (fl.), *Senna s. n.* (*Herb. Schwacke 11747*) (NY, P, RB); Lagoa Santa, s. d. (fl.), *E. Warming s. n.* (P).

#### Recognition and notes.

*Rourea cnestidifolia* is recognised by the presence of glandular trichomes, relatively large middle and apical leaflets (4–7.2 × 1.6–3.2 cm), obtuse, acute or narrowly-rounded leaflet apex, a long peduncle (2.8–8 cm long) and a short pedicel (3–5 mm long).

The morphological limits separating *Rourea cnestidifolia* and *R. glazioui* present slight discontinuities, but these, along with distribution patterns, are sufficient to distinguish them. They are similar in the presence of glandular trichomes on petiole, leaf rachis and inflorescences, leaflet size and shape and in overall characteristics of flowers and fruits. Schellenberg (1938) separated them based on pedicel length, while Forero (1976, 1983) used number of leaflets. These are useful distinctions despite some overlapping characters, so this revision considers that *R. cnestidifolia* differs from *R. glazioui* by the leaves 9–13-foliolate (Fig. 1D), peduncle 2.8–8 cm long, flowers congested in the inflorescence apex and pedicel 3–5 mm long vs. leaves 13–27-foliolate (Fig. 10), peduncle 0.2–1.7 cm long, flowers loosely disposed in the inflorescences and pedicel 5–10(–14) mm long. Additionally, the indumentum of branchlets and leaflets (lower surface) is denser in *R. glazioui* than it is in *R. cnestidifolia*. Geographic distribution may also be useful for recognition: *R. cnestidifolia* is apparently restricted to central and northeast portions of Minas Gerais, while *R. glazioui* is very common in the coastal zone between southern Bahia and central Rio de Janeiro (Fig. 6).

Forero (1976) selected the lectotype of *R. cnestidifolia*, as the type from B is considered missing. The specimen indicated by Forero (1976) from K has no collection date, although the author cited “18 Nov 1864”, probably because he considered it the same collection of specimen Warming 1849/3 from C (barcode C 10009584) and Warming 1849/1 from GH (barcode GH 00043365). However, there are, in C, many specimens Warming 1849, in which collection number is subdivided from 1 to 5 and present different collection dates. All specimens of Warming 1849 from C, GH and K seem to correspond to the same gathering, but as they do not match in collection dates and subdivision of main collection number, the lectotype from K is here considered a unicate.

### Rourea diamantina

Taxon classificationPlantaeOxalidalesConnaraceae

C. Toledo sp. nov.

02245C4B-B280-5C98-8041-758EADC92D00

urn:lsid:ipni.org:names:77213227-1

Fig. 7

#### Type.

**Brazil. Bahia**: Itatim, interior da mata da base do Inselberg, 12°45'12"S, 39°46'59"W, 26 Jan 1997 (fl.), *E. Melo et al. 1985* (***Holotype***: ESA 84255!; ***isotypes***: HUEFS!, UEC!, VIC!).

#### Diagnosis.

Akin to *R. martiana* due to the presence of glandular trichomes, relatively small leaves and leaflets and flowers and fruits with similar characteristics, but differs by the leaves 5–9(–13)-foliolate (vs. 9–15-foliolate), leaflet apices narrowly rounded or obtuse (vs. rounded) and pedicels 5–13 mm long (vs. ca. 2 mm long).

#### Description.

***Lianas***, shrubs or scandent shrubs, rarely treelets, 3–4 m tall; branchlets subglabrous or sparsely pubescent, lenticels abundant, conspicuous. ***Leaves*** 5–9(–13)-foliolate, congested; petiole 1.3–2.2 cm long, sparsely hirsute to glabrescent, eglandular; rachis 3–6(–8.5) cm long, sparsely hirsute to glabrescent, eglandular; ***leaflets*** opposite to subopposite, pulvinulus ca. 1 mm long; blade of the basal pair of leaflets 1.4–2 × 0.9–1.4 cm, ovate, others 2–4.5(–5.8) × (0.8–)1.2–1.7(–2.2) cm, narrowly ovate, rarely oblong, chartaceous, occasionally membranaceous, slightly discolorous, abaxially hirsute or villous, brownish or greenish, adaxially subglabrous, dull, base slightly asymmetric, rounded or subcordate, rarely obtuse, apex narrowly rounded or obtuse, margin flat or slightly revolute, ciliate; midvein abaxially prominent, adaxially flat or slightly impressed, secondary veins 5–7 pairs, abaxially slightly prominent, adaxially flat, tertiary veins abaxially slightly prominent, adaxially flat or slightly prominent. ***Inflorescences*** in pseudoterminal cymes, rarely ramiflorous; bracts 1.5–2.5 mm long; peduncle 0.8–1.7 cm long, sparsely hirsute, eglandular; rachis 1–2.5 cm long, sparsely hirsute, eglandular. ***Flowers*** loosely disposed; buds not seen; pedicel 5–13 mm long, with glandular trichomes, 2 bracteoles located up the lower half, persistent; sepals 4.5–5 × 2–3 mm, chartaceous, ovate, outer surface pubescent, with glandular trichomes, inner surface sericeous, margin ciliate; petals 7–8 × 2.5–3 mm, narrowly obovate, glabrous on both surfaces; stamens connate at base by ca. 1 mm, shorter series 2–3 mm long, longer series 3–4 mm long, glabrous; ovary ca. 1 mm long, densely hirsute, style ca. 4 mm long, sparsely hirsute, stigma peltate, bilobate. ***Fruits*** 1.1–1.4 × 0.6–0.7 cm, yellowish or orangish, outer surface subglabrous, sparsely hirsute at the apex, inner surface subglabrous, apex acuminate, style partially persistent, calyx covering one third of the fruit; ***seeds*** 0.7–0.9 × 0.4–0.5 cm, arillode colour not seen.

**Figure 7.**
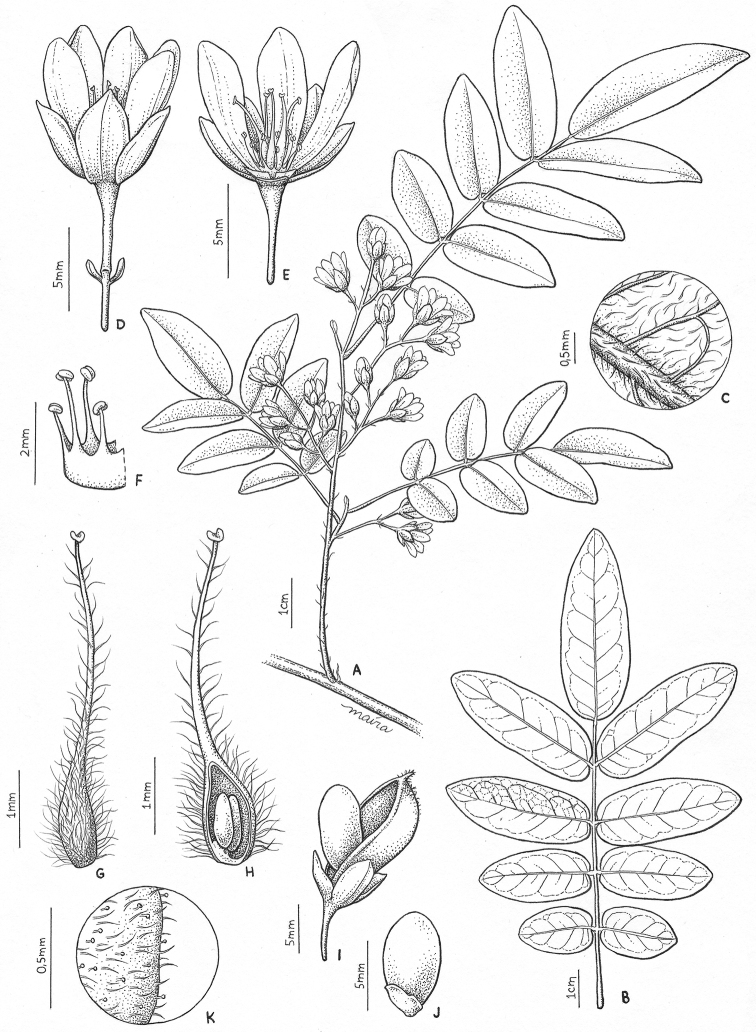
*Rourea diamantina*: **A** flowering branchlet **B** leaf, abaxial surface **C** indumentum, leaflet abaxial surface **D** flower, external view **E** flower, internal view **F** stamens **G** ovary, external view **H** ovary, internal view and ovules **I** fruit and seed, external view **J** seed, external view **K** sepal indumentum, external view.

#### Distribution, habitat and phenology.

**Figure 8.**
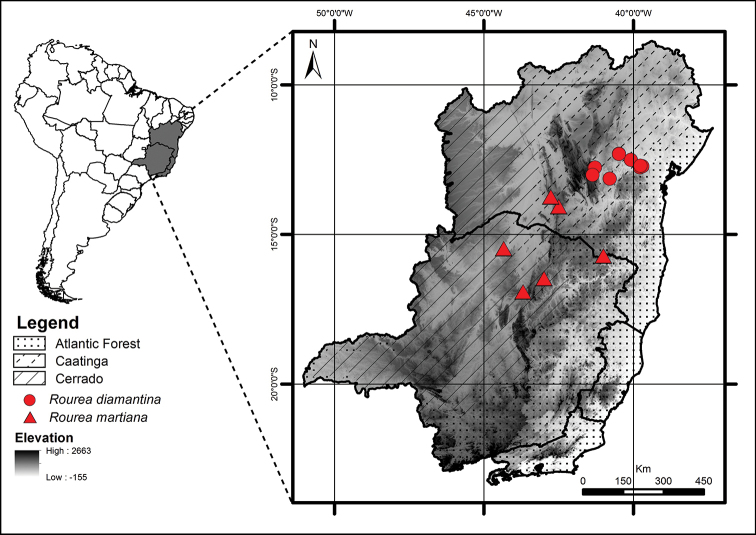
Geographic distribution of *Rourea diamantina* (circles) and *R. martiana* (triangles).

*Rourea diamantina* is only known from the east side of Chapada Diamantina, a mountain range of about 41,700 km^2^ and approximately 2,000 m altitude, located in the centre of Bahia (Fig. 8). Individuals of the new species are mostly shrubs with scandent branches, occurring in seasonal forests of Inselbergs. Specimens have been collected with flowers from September to December and with fruits from November to February and in July.

#### Paratypes.

**Brazil. Bahia**: Estrada para o Riacho do Meio, 12°18'18"S, 40°29'11"W, 07 Feb 2005 (fr.), *D. Cardoso 242* (CEPEC, HUEFS, NY); Itaberaba, Fazenda Itaberaba, ilhas de vegetação em Inselbergs, 12°30'06"S, 40°05'03"W, 08 Feb 2007 (fr.), *J. L. Ferreira et al. 301* (HUEFS); Morro do Agenor, 12°42'S, 39°46'W, 26 Nov 1995 (fl.), *F. França et al. 1482* (HUEFS); Morro das Tocas: 12°43'S, 39°42'W, 28 Sep 1996 (fl.), *F. França et al. 1844* (ALCB, HUEFS, SPF); 29 Sep 1996 (fl.), *F. França et al. 1866* (HUEFS, SPF). Mucugê, Chapada Diamantina, caminho para Marimbus, 14 Dec 2013 (fr.), *M. K. Guedes et al. 21064* (ALCB); Chapada Diamantina, Marimbus, 12°76'30"S, 41°30'91"W, 14 Nov 2014 (fr.), *M. K. Guedes et al. 23112* (ALCB, MBML); Rodovia BR 116, Feira de Santana, Milagres, 12°43'S, 39°42'W, 24 Nov 2001 (fr.), *J. G. Jardim 3973* (CEPEC, HUEFS); Morro do Agenor ou da Madeira, 12°43'S, 39°42'W, 17 Dec 1995 (fl.), *E. Melo et al. 1409* (ALCB, HUEFS); Morro da Torre, 12°43'S, 39°42'W, 09 Nov 1996 (fr.), *E. Melo et al. 1832* (ESA, HUEFS, RB); Itatim, interior da mata da base do Inselberg, 12°45'12"S, 39°46'59"W. 26 Jan 1997 (fr.), *E. Melo et al. 1981* (ESA, HUEFS, UEC); Andarai, Alagados Marimbus, 12°45'55"S, 41°18'52"W, 07 Dec 2012 (fr.), *E. Melo et al. 11815* (HUEFS); Rui Barbosa, Serra do Orobó, base da encosta da serra, 12°18'S, 40°29'W, 18 Dec 2004 (fl.), *L. P. de Queiroz et al. 9894* (HUEFS); Machado Portello, 23 Jul 1915 (fr.), *J. N. Rose & P. G. Russel 19932* (NY, US); Cachoeira, 03 Jan 1977 (fr.), *P. de Souza s. n.* (ALCB, CEPEC).

#### Etymology.

The specific epithet “diamantina” refers to Chapada Diamantina (Bahia, Brazil), where the new species is presumed to be endemic. This epithet is a noun in apposition (Turland et al. 2018, Art. 23.5).

#### Recognition and notes.

*Rourea diamantina* is recognised by the leaves 5–9(–13)-foliolate, leaflets abaxially hirsute or villous, pedicel with glandular trichomes and sepals with indumentum sericeous internally. An interesting characteristic is the leaves whose leaflets become significantly larger towards the apex. The new species is similar to *R. martiana*, but differs by the reduced number of leaflets (usually 5–9), which are normally narrowly ovate with obtuse or narrowly-rounded apex, and longer pedicel (5–13 mm long) vs. leaves 9–15-foliolate, leaflets normally oblong or narrowly elliptic with rounded apex and a shorter pedicel (ca. 2 mm long). Additionally, both species are geographically isolated by the Chapada Diamantina (Fig. 8).

Some specimens, cited here under *Rourea diamantina*, were previously identified as *R. martiana* by Schellenberg (1938) and Forero (1976, 1983). After analysing modern collections from Bahia and Minas Gerais, this revision considers that the morphological differences indicated above and their disjunct distribution provide evidence to recognise *R. diamantina* apart from *R. martiana*. For a complete discussion on this subject, see “Recognition and notes” section of *R. martiana*.

### Rourea discolor

Taxon classificationPlantaeOxalidalesConnaraceae

Baker, in Martius, Fl. Bras. 14(2): 180. 1871.

D4FF7438-4032-5965-A758-A6BF60EBC7F9

Fig. 1A

Santalodes discolor (Baker) Kuntze, Revis. Gen. Pl. 1: 155. 1891.
Eichleria lucida Progel, in Martius Fl. Bras. 12(2): 1877. Rourea progeliana Kuhlm., Arq. Inst. Biol. Veg. 1: 40. 1934. Type. Brazil. Bahia: S. d. (fl.), *J. S. Blanchet 3145A* (*Lectotype*: P barcode P 02274093!, designated by Forero 1983; *isolectotypes*: G!, OXF, W!).

#### Type.

**Brazil. Bahia**: Ilheos, 04 Sep 1839 (fl.), *B. Luschnath s. n.* (***Holotype***: BR barcode BR 697465!).

#### Description.

***Lianas*** or scandent shrubs, 2–3 m tall; branchlets glabrous, lenticels sparse or abundant, conspicuous or inconspicuous. ***Leaves*** 9–25(–29)-foliolate, loosely disposed; petiole 1.7–4 cm long, glabrous or subglabrous, rarely sparsely villous, eglandular; rachis 6.7–12.5(–18) cm long, glabrous or subglabrous, rarely sparsely villous, eglandular; ***leaflets*** opposite to subopposite, pulvinulus 1–2 mm long; blade of the basal pair of leaflets 1.3–2.7(–3.3) × 0.8–1.6 cm, elliptic, narrowly elliptic, oblong or narrowly ovate, others 1.8–4.5(–5.8) × 0.8–1.7(–2.3) cm, narrowly elliptic, oblong or narrowly obovate, apical ones usually elliptic, chartaceous or subcoriaceous, discolorous, abaxially glabrous, sparsely puberulous, rarely pubescent, glaucous, occasionally greenish, adaxially glabrous, dull or shinning, base symmetric or slightly asymmetric, cordate, subcordate, occasionally truncate, sometimes obtuse in the apical leaflet, apex rounded, occasionally slightly obtuse, margin flat or revolute, glabrous; midvein abaxially prominent, adaxially flat, secondary veins 6–8 pairs, abaxially flat, adaxially slightly prominent, tertiary veins abaxially flat, adaxially slightly prominent. ***Inflorescences*** in axillary or pseudoterminal cymes or panicles; bracts ca. 1 mm long; peduncle 0.2–2.7 cm long, glabrous or subglabrous, eglandular; rachis 3.5–11.5 cm long, glabrous or subglabrous, eglandular; lateral branches 2.2–9.5 cm long, glabrous or subglabrous, eglandular. ***Flowers*** loosely disposed; buds 2–3 × 2–3 mm, orbicular or ovate; pedicel 6–16 mm long, eglandular, 1–2 bracteoles located up the lower half, deciduous; sepals (3.5–)4.5–5.5 × 2–2.5 mm, chartaceous, ovate, outer surface glabrous, pubescent only at the apex, eglandular, inner surface glabrous, margin glabrous or ciliate; petals (4–)6–8 × 2–2.5 mm, narrowly obovate, glabrous on both surfaces; stamens connate at base by ca. 1 mm, shorter series ca. 2.5 mm long, longer series ca. 3.5 mm long, glabrous; ovary ca. 1 mm long, densely hirsute, style ca. 1 mm long, sparsely hirsute, stigma peltate, bilobate. ***Fruits*** 1.2–1.7 × 0.4–0.6 cm, orangish or reddish, outer surface glabrous or subglabrous, sparsely hirsute only at the apex, inner surface glabrous, apex acuminate, style partially persistent or deciduous, calyx covering one third of the fruit; ***seeds*** ca. 1.3 × 0.5 cm, arillode yellowish.

#### Distribution, habitat and phenology.

**Figure 9.**
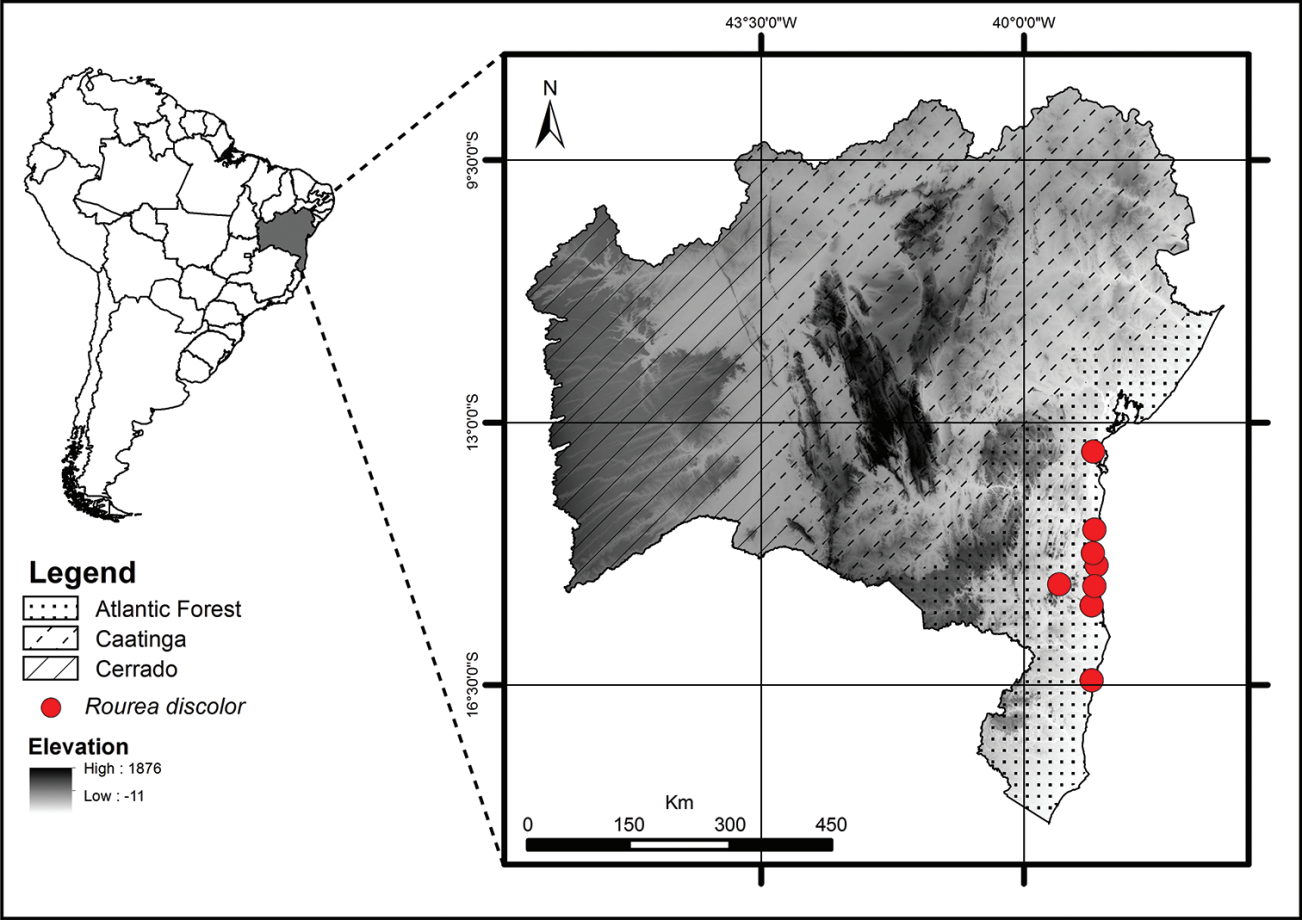
Geographic distribution of *Rourea discolor*.

*Rourea discolor* is exclusive to Bahia, where the individuals are distributed mainly in the southern coastal zone (Fig. 9). Its limits range from the municipality of Valença (near Itacaré) to the municipality of Porto Seguro. This is a lianescent species up to 3 m tall and occurs in dense ombrophilous, “Tabuleiro” or swamp forests, sometimes reaching areas of restinga (coastal vegetation), growing on clay or sandy soils. Specimens have been collected with flowers from August to September and with fruits from October to February.

#### Specimens examined.

**Brazil. Bahia**: Estrada que liga Serra Grande/Itacaré, coletas a 8 km partindo da Serra Grande, 26 Aug 1992 (fl.), *A. M. Amorim 671* (CEPEC, HUEFS, NY); Reserva Biológica do Mico-Leão (IBAMA), estrada no km 48 da Rod. BA-001 Ilhéus/Una, região da mata higrófila Sul Baiana, 15°09'S, 39°05'W, 18 Aug 1997 (st.), *A. M. Amorim et al. 2069* (CEPEC); Jussari, RPPN Serra do Teimoso, rodovia Jussari-Palmira, estrada ca. 7.5 km de Jussari, 15°09'16"S, 39°31'52"W, *A. M. Amorim et al. 4135* (CEPEC); 10 km S de Pontal (Ilhéus), camino a Olivença, local de extraccion de arena, aprox. 14°54'S, 39°02'W, 04 Dec 1992 (fr.), *M. M. Arbo et al. 5559* (SPF); Reserva Biológica de Una, estrada no km 46 da Rodovia BA-001, Ilhéus/Una, estrada principal para a sede, trilha piedade, 15°09'S, 39°05'W, 10 Sep 2006 (fl.), *A. M. Amorim et al. 6274* (CEPEC, NY, SPF); Valença, estrada para Orobó, com entrada no km 3 da estrada Valença/BR 101, coletas entre o km 3–10 do ramal para Orobó, 07 Feb 1983 (fr.), *A. M. de Carvalho & T. Plowan 1506* (CEPEC); Distrito de Serra Grande, 7.3 km na estrada Serra Grande/Itacaré, Fazenda Lagoa do Conjunto Fazenda Santa Cruz, 14°25'S, 30°01'W, 11 Nov 1991 (fl.), *A. M. de Carvalho et al. 3512* (ALCB, CEPEC, HUEFS, NY, US); Olivença, mata do Balneário Tararomba, 15°26'S, 39°06'W, 17 Sep 2016 (fl.), *M. L. Guedes et al. 24980* (ALCB); Rod. BA-001 Ilhéus/Una ca. 50,5 km de Ilhéus, área de influência da REBIO de Una, 12 Aug 2000 (fl.), *J. G. Jardim et al. 3094* (CEPEC, NY, RB); Distrito de Serra Grande, Faz. Lagoa do Conjunto e Conjunto Faz. Santa Cruz, ramal que dá acesso à sede da fazenda, ca. 300 m da rodovia, 07 Jan 2000 (fr.), *F. do S. Juchum et al. 09* (CEPEC, NY); Porto Seguro, 02 Sep 1961 (fl.), *A. P. Duarte 6118* (NY, RB, US); Una, Reserva Biológica da Una (REBIO da Una), estrada ca. 45 km S de Ilhéus, margem da estrada que lava à sede da reserva, 15°10'46"S, 39°03'50"W, Jan 2001 (fr.), *F. do S. Juchum et al. 117* (CEPEC, NY); Rod. Una-Camandatuba, mata litorânea, solo arenoso na Faz. De Antônio Pimenta, 26 Oct 1971 (fr.), *R. S. Pinheiro 1656* (CEPEC, NY); Ilhéus, Sep 1821 (fl.), *L. Riedel s. n.* (NY barcode NY 00393556); Serra Grande, 25 Aug 1996 (fl.), *A. L. B. Sartori et al. 293* (CEPEC, UEC); 3 km north of Rodoviaria, Mata da Esperança, forest north of dam and reservoir, 14°46'55"S, 39°04'09"W, 20 Sep 1994 (fl.), *W. W. Thomas et al. 10574* (MBM, NY); Uruçuca, 7.3 km north of Serra Grande on road to Itacaré, Fazenda Lagoa do Conjunto Fazenda Santa Cruz, 14°25'24"S, 39°03'38"W, 15 Nov 1995 (fr.), *W. W. Thomas et al. 11015* (CEPEC).

#### Recognition and notes.

*Rourea discolor* is recognised by the glabrous or subglabrous inflorescence rachis measuring 3.5–11.5 cm long and pedicel 6–16 mm long. It is morphologically similar to *R. glazioui* due to the number and shape of its leaflets; however, individuals of *R. discolor* do not have glandular trichomes and the inflorescence rachis is glabrous or subglabrous, while in *R. glazioui*, they have glandular trichomes and the inflorescence rachis is hirsute or densely so. *Rourea discolor* can be confused with *R. bahiensis* as well, although they are differentiated by the characteristics of the leaflets and inflorescences (see “Recognition and notes” section of *R. bahiensis*).

In the original description, Baker (1871) indicated only the specimen *Luschnath s. n.*, without mentioning either herbarium, collector number or date. This specimen is deposited in BR and is likely to be the holotype, as no other duplicate has been found.

*Rourea discolor* was once treated in Oxalidaceae by Progel (1877) under *Eichleria lucida*. Kuhlmann (1934) proposed the combination of *Eichleria* in *Rourea*, but the basionym *Rourea lucida* already existed, so he named it as *R. progeliana*. This name is, however, a heterotypic synonym of *R. discolor*, as the latter had been published previously (Baker 1871).

### Rourea glazioui

Taxon classificationPlantaeOxalidalesConnaraceae

G. Schellenb., in Engler, Pflanzenreich IV. 127(Heft 103): 289. 1938.

9109CE74-9145-5751-9F87-A65F94FB25D7

Fig. 10

Rourea polyphylla G. Schellenb., in Engler, Pflanzenreich IV. 127(Heft 103): 197. 1938, *nom. illeg*., non R. polyphyllaBlume (1849).

#### Type.

**Brazil. Rio de Janeiro**: Rezende, 22 Nov 1876 (fr.), *A. F. M. Glaziou 8625* (***Holotype***: B†; ***lectotype***: P barcode P 02274098!, selected by Forero 1976; ***isolectotypes***: C!, F-frag., K!).

#### Description.

***Lianas*** or scandent shrubs, 0.9–1.5 m tall; branchlets densely velutinous to glabrescent, lenticels sparse or abundant, conspicuous or inconspicuous. ***Leaves*** (9–)15–27-foliolate, loosely disposed; petiole 1.9–3.5(–4.5) cm long, densely velutinous or hirsute, with glandular trichomes; rachis (8–)10–24 cm long, densely velutinous or hirsute, with glandular trichomes; ***leaflets*** opposite to alternate, subsessile or pulvinulus ca. 1 mm long; blade of the basal pair of leaflets 1.3–3.3 × (0.8–) 1.2–2.6 cm, ovate, oblong or orbicular, rarely elliptic, others (1.5–)2.6–6.5(–8.2) × (0.8–) 1.3–2.6 cm long, narrowly ovate, narrowly obovate, oblong or narrowly elliptic, rarely elliptic, apical ones usually elliptic, chartaceous, discolorous, abaxially hirsute to densely hirsute, greenish or brownish, adaxially subglabrous to sparsely hirsute, more densely on midvein, dull, base slightly asymmetric to asymmetric, very rarely symmetric, rounded, subcordate, cordate or truncate, rarely obtuse, occasionally acute in the apical leaflet, apex narrowly rounded or obtuse, rarely rounded, occasionally acute in the apical leaflet, margin slightly revolute to revolute, rarely flat, ciliate; midvein abaxially prominent, adaxially slightly impressed, occasionally flat, secondary veins 6–8(–9) pairs, abaxially prominent or slightly prominent, rarely flat, adaxially slightly impressed or flat, tertiary veins abaxially slightly prominent or flat, adaxially slightly impressed or flat. ***Inflorescences*** in axillary cymes, rarely panicles; bracts 2–3 mm long; peduncle 0.2–1.7 cm long, hirsute to densely hirsute, with glandular trichomes; rachis 0.3–4(–8) cm long, hirsute to densely hirsute, with glandular trichomes; lateral branches 0.3–1.5(–2.8) cm long, hirsute to densely hirsute, with glandular trichomes. ***Flowers*** loosely disposed; buds 3–4 × 2–3 mm, ovate, orbicular or ellipsoid; pedicel 5–10(–14) mm long, with glandular trichomes, 1–2 bracteoles located up the lower third, deciduous or persistent; sepals (4–)4.5–5.5 × 1.5–2 mm, chartaceous, ovate or elliptic, outer surface hirsute or sparsely hirsute, with glandular trichomes, inner surface sericeous or sparsely sericeous, margin ciliate; petals (5–)6–7.5 × 1.5–2 mm, narrowly obovate or oblong, glabrous on both surfaces; stamens connate at base by 0.8–1 mm, shorter series 2.5–4 mm long, longer series 4–5 mm long, glabrous; ovary 1–1.2 mm long, densely hirsute, style (1.5–)4–5.5 mm long, sparsely hirsute, subglabrous or glabrous only at the apex, stigma peltate, bilobate. ***Fruits*** 1.1–1.5(–1.6) × 0.5–0.6(–0.8) cm, orangish, reddish or yellowish, outer surface partially or completely hirsute, usually more densely at the apex, inner surface glabrous or subglabrous, apex acuminate or rounded, style partially persistent or deciduous, calyx covering one third of the fruit; ***seeds*** 0.9–1.1(–1.3) × 0.3–0.5(–0.6) cm, arillode yellowish.

#### Distribution, habitat and phenology.

*Rourea glazioui* is found in Bahia, Espírito Santo and Rio de Janeiro (Fig. 6). In ES, the species has been widely collected along the central and east parts of the state, whereas in BA it is restricted to the southern region, and in RJ, it is sparsely distributed in the eastern side. The type location (Rezende, RJ) might be mistaken as no other record has been found nearby. It is a liana or scandent shrub up to 1.5 m tall, mainly occurring in areas of ombrophilous or “Tabuleiro” forests, although sometimes found in swamp forests or disturbed environments, such as small fragments or in *Eucalyptus* plantations, growing on clay or sandy soils. Specimens have been collected with flowers and fruits almost throughout the year, although more frequently during the spring season.

#### Specimens examined.

**Brazil. Bahia**: Nova Viçosa, ca. 61 km na estrada de Caravelas para Nanuque, 06 Sep 1989 (fr.), *A. M. de Carvalho et al. 2499* (CEPEC, MBM); Picadão, extremo Sul, área da Aracruz Celulose, 23 Aug 1993 (fr.), *M. L. Guedes 2971* (ALCB, CEPEC); Caravelas, área de influência da CAF, 17°44'07"S, 39°45'16"W, 03 Feb 2002 (fr.), *M. L. Guedes 9705* (ALCB); Teixeira de Freitas. BR-101 ca. 11 km da cidade, 17°25'49"S, 39°41'14"W, 18 Jun 2005 (fl.), *J. G. Jardim et al. 4620* (CEPEC, HUEFS); Assentamento “Paulo Freire” (MST), ramal com entrada no km 15 da Rodovia Macuri/Itabatan, 2 km antes da sede do assentamento, 04 Oct 2000 (fr.), *L. A. Matos-Silva et al. 4140* (ALCB, CEPEC, HUEFS, NY, UESC); Mucuri, 14–17 km a W de Mucuri, 13 Sep 1978 (fl.), *S. A. Mori et al. 10436* (CEPEC, NY); Macuri, área de restinga com algumas manchas de campos, a 7 km a NW de Macuri, 14 Sep 1978 (fl., fr.), *S. A. Mori et al. 10532* (CEPEC, NY); Nova Viçosa, 3 km após posto da mata, sentido Posta da Mata divisa BA-MG, 08 Nov 1999 (fr.), *A. A. Santos et al. 553* (CEN); Vale do Rio Alcobaça, 12 May 1971 (fl.), *T. S. dos Santos 1613* (CEPEC, US). **Espírito Santo**: Boa Esperança, Bela Vista, 18°33'21"S, 40°13'10"W, 115 m alt., 1 Dec 2010 (fr.), *A. M. Assis & M. D. S. Demuner 2608* (MBML); Sooretama, REBIO Sooretama, Quirinho, 19°03'14"S, 40°09'35"W, 80 m alt., 2 Nov 2013 (fl.), *A. M. Assis et al. 4030* (VIES); Guarapari, Barro Branco, próximo da BR 101, 20°33'11"S, 40°28'39"W, 17 Jul 2018 (fr.), *A. M. Assis et al. 4516* (VIES); Reserva Fazenda São Joaquim, 14 Oct 1985 (fr.), *H. Q. Boudet & W. Boone 2027* (MBM, RB); Pinheiros, Reserva Biológica do Córrego do Veado, trilha que vai para mata de água limpa, 09 Jul 2010 (fr.), *I. S. Broggio 26* (VIES); Guarapari, Parque Natural Municipal Morro da Pescaria, 20.6591S, 40.4731W, 09 Feb 2014 (fl.), *A. C. S. Dal Col & J. Rodrigues Filho 265* (VIES); Governador Lindemberg, Mata da Prefeitura, 14 Nov 2006 (fr.), *V. Demuner et al. 3069* (MBM, UB); Conceição da Barra. 16 Aug 1965 (fl., fr.), *A. P. Duarte 8888* (NY, RB); Entre Linhares e São Matheus, 4 Nov 1953 (fr.), *A. P. Duarte & J. C. Gomes 3960* (RB); Comunidade de Lajinha, Fazenda Rancho Tropical II, restinga arbustiva alta com moitas, 05 Jul 2007 (fl., fr.), *C. Farney et al. 4764* (RB); Pedro Canário, estradas vicinais, próximas ao eixo da BR 101 entre o Rio Itaúnas e 5 km em direção a Pinheiro, 21 Oct 2008 (fr.), *C. Farney et al. 4881* (RB); Reserva Biológica do Córrego Grande, estrada no meio da reserva, 28 Dec 2012 (fr.), *T. B. Flores & G. O. Romão 1257* (ESA, RB); Floresta Nacional do Rio Preto, trilha da Lagoa Seca, 29 Aug 2012 (fr.), *T. B. Flores & G. O. Romão 1284* (ESA); Presidente Kennedy, 6 Feb 1988 (fl.), *J. M. L. Gomes 476* (VIES); Próximo à antiga casa do guarda, estrada Aderne, 04 Jul 1995 (fl.), *D. A. Folli 2342* (CEPEC, CVRD, ESA); Próximo ao Rio Barra Seca, estrada Aderne, 06 Oct 1994 (fr.), *D. A. Folli 2385* (CEPEC, CVRD, ESA); Jueirana, estrada Aceiro com Eucalipto, 25 Jul 2001 (fl.), *D. A. Folli 3996* (CVRD, ESA); BR 101, próximo à entrada para Conc. da Barra, 27 Aug 2007 (fl.), *D. A. Folli 5684* (CVRD, ESA); Mata de restinga sobre feixes de cordões arenosos, vegetação localizada à direita da estrada principal da vila de Itúnas, 06 Oct 2007 (fr.), *A. O. Giaretta et al. 248* (RB); Vegetação de restinga, mata seca, entrada localizada adjacente à estrada principal da Vila de Itaúnas, área de preservação permanente à PEI, 14 Jun 2008 (fr.), *A. O. Giaretta et al. 276* (RB); Jaguaré, Rod. BR-101, 23 Aug 1987 (fr.), *G. Hatschbach & A. C. Cervi 51416* (MBM, US); Rod. ES-421, km 5–8, 09 Oct 1998 (fr.), *G. Hatschbach et al. 68346* (CEPEC, MBM, US); Itaúnas, 09 Jun 1992 (fl.), *O. J. Pereira* 3419 (VIES); 20 May 1999 (fl.), *G. Hatschbach et al. 69202* (CEPEC, ESA, MBM, SPF, US); Praia Setibana, ES-060 at 6 km E of BR-101, 18 Jan 1993 (fr.), *J. A. Kallunki & J. R. Pirani 345* (NY, SPF); Colatina, estrada do Patrimônio, perto de Colatina, 16 May 1934 (fl.), *J. G. Kuhlmann 351* (IAN, NY, RB); Reserva Biológica do Córrego Grande, coletado próximo na mata próxima a sede da rebio, 10 Jan 2012 (fr.), *L. Marcarini et al. 38* (VIES); Próximo ao Bairro Litorâneo, seguindo uma estrada de terra atrás do campus da Universidade (UFES), 01 Aug 2007 (fr.), *R. F. A. Martins* et al. *38* (RB); Rodovia do Sol, road linking BR-101 to the São Mateus, Bairro Litorâneo, fragmento de mata ciliar próximo ao campus da universidade, 03 Oct 2009 (fr.), *A. G. Oliveira & M. Ribeiro 656* (VIES); Anchieta, Estrada para Castellanos, tipo do morro, 2 Feb 2012 (fl.), *N. E. Oliveira Filho 79* (VIES); Mata seca de restinga, 23 Jun 2002 (fl.), *O. J. Pereira et al. 3536* (VIES); Área 126 da Aracruz Celulose S. A., 2 Apr 1992 (fr.), *O. J. Pereira 4283* (VIES); Serra, Bicanga, 22 Apr 1993 (fl.), *O. J. Pereira 4529* (VIES); Itaúnas, área 135 da Aracruz Celulose S. A., 18°25'10"S, 39°42'32"W, 21 Sep 1993 (fr.), *O. J. Pereira et al. 4890* (VIES); Interlagos, Rodovia do Sol ES060, 20°19'47"S, 40°17'32"W, 1 Jun 1995 (fr.), *O. J. Pereira 5468* (VIES); Itaúnas, 13 Jul 1991 (fr.), *P. C. Vinha 1271* (VIES); Linharinho, 6 Nov 1996 (fr.), *O. J. Pereira et al. 5726* (VIES); Vila Velha. 20°25'42.7"S, 40°22'46.7"W, 10 Jan 2001 (fl.), *O. J. Pereira & E. Espindula 6713* (VIES); Parque Estadual de Itaúnas, 24 Aug 2002 (fr.), *O. J. Pereira et al. 6983* (VIES); Serra, Nova Zelândia, 20°10'52"S, 40°12'54"W, 21 Jul 2015 (fr.), *O. J. Pereira et al. 8021* (VIES); Linhares, Reserva Natural Vale, aceiro c/ BR 101 jueirana, 21 Aug 2006 (fr.), *G. S. Siqueira 240* (CVRD, ESA); Parque Estadual de Itaúnas, Trilha Alméscar, 18.4033S, 39.7019W, 08 Aug 2013 (fr.), *W. O. Souza et al. 146* (VIES); Conceição da Barra, Itaúnas, área da Fíbria com plantação de eucalipto, 18°29'27"S, 39°44'12"W, 21 Oct 2018 (fr.), *C. A. P. Toledo & N. C. Bígio 400* (ESA); Guarapari, Parque Estadual Paulo César Vinha, 21 Oct 2006 (fr.), *R. T. Valadares 304* (VIES); Aracruz, Estação Biológica Marinha Augusto Ruschi, Santa Cruz, 19°58'14"S, 40°08'26"W, 1 Apr 2018 (fr.), *Wandekoken et al. 257* (VIES); Parque Ecológico da CST, área de Tabuleiro, Bosque dos Jacarandás, área dominada por espécies exóticas plantadas, 21 Apr 1995 (fl.), *I. Weiler Junior et al. 166* (VIES). **Rio de Janeiro**: Casimiro de Abreu, Morro de S. João, 03 Feb 1970 (fr.), *CPJ s. n.* (RB 261147); Reserva Biológica do Poço das Antas, mata da Osmarina, 26 May 1982 (fr.), *H. C. de Lima & G. Martinelli 1733* (RB); Silva Jardim, Est. Juturnaiba, esquerda – km 5, Reserva Biológica de Poço das Antas, 11 Jan 1994 (fr.), *C. Luchiari et al. 349* (RB); Conceição de Macabu, km 12 da BR 101, a 62 km de Campos, 10 Jan 1985 (fr.), *J. R. Pirani & D. C. Zappi 1046* (NY, SPF); Horto Florestal de Rezende, s. d. (st.), *A. da Silva s. n.* (IAN 67552).

#### Recognition and notes.

*Rourea glazioui* resembles *R. cnestidifolia* as they have glandular trichomes and similar characteristics of leaflet shape and size, flowers and fruits. However, the former has leaves (9–)15–27-foliolate, peduncle 0.2–1.5 cm long, flowers loosely disposed on the inflorescences and pedicel 5–10(–14) mm long, while the latter has leaves 9–13-foliolate, peduncle 2.8–8 cm long, flowers congested in the inflorescence apex and pedicel 3–5 mm long. *Rourea glazioui* is commonly confused with *R. chrysomalla* in herbarium specimens, but differs in the characteristics described in the “Recognition and notes” section of *R. chrysomalla*.

In the protologue, *Rourea glazioui* was named as *R. polyphylla* (Schellenberg 1938). After noticing that he created a homonym of *R. polyphylla*Blume (1849), Schellenberg (1938) added an appendix in the same work and replaced *R. polyphylla* for *R. glazioui*. This does not preclude valid publication of *R. glazioui*, as this replaced name, although indicated in the same work, presented a clear and crossed reference of a corresponding description (Turland et al. 2018, Art. 41.3).

The type collection of *R. glazioui* morphologically resembles the type of *R. fulgens* Planch. (*Wallich 8524*, deposited in K), a species restricted to Singapore. Both type specimens share densely-velutinous branchlets, multifoliolate leaves, and oblong or narrowly-elliptic leaflets with rounded apex, which are also discolorous and abaxially hirsute. Some other species from south-eastern Asia, such as *R. mimosoides* (Vahl) Planch., also have multifoliolate leaves with oblong or narrowly-elliptic leaflets, so this raises the question whether *Rourea* species from the New World are a monophyletic group. Morphological similarities between the multifoliolate species of *Rourea* from America and south-eastern Asia were firstly noticed by Forero (1976), who cited R. sect. Mimosoideae Planch. *p. p.* under R. subgen. R. sect. Multifoliolatae, thus drawing attention to possible relationships. Nevertheless, this subject should be addressed by future molecular investigations.

**Figure 10.**
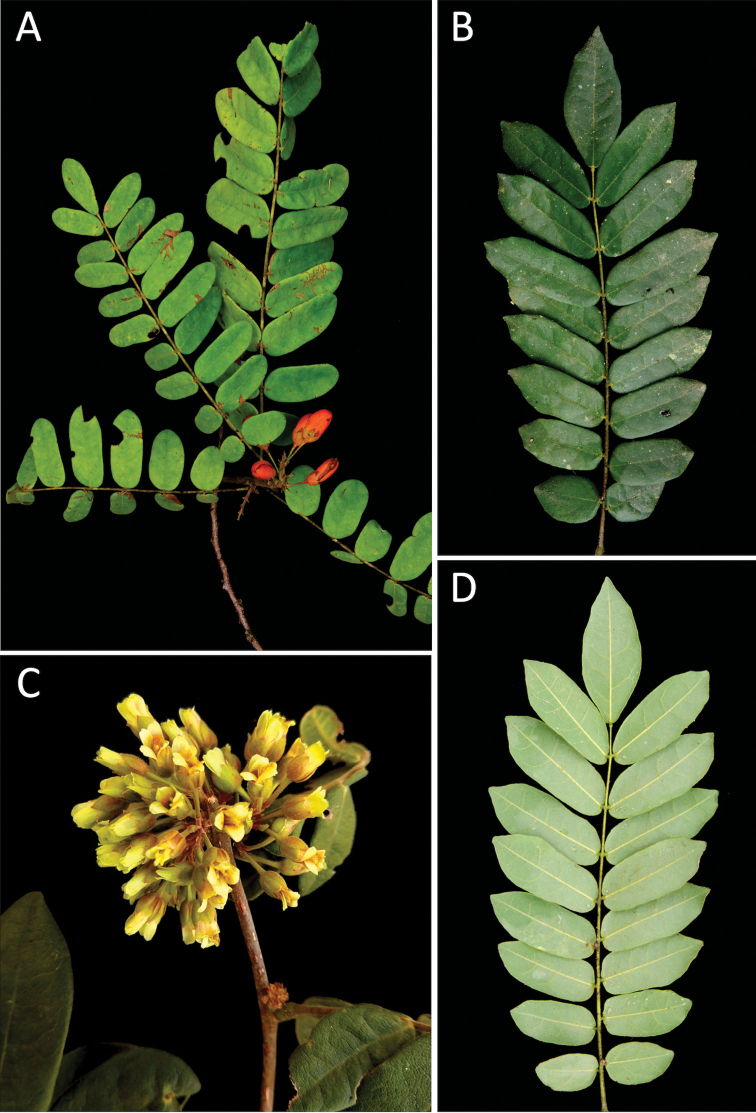
*Rourea glazioui*: **A** fruiting branchlet **B** leaf, adaxial surface **C** inflorescence (photo by Thiago Flores) **D** leaf, abaxial surface.

### Rourea macrocalyx

Taxon classificationPlantaeOxalidalesConnaraceae

Carbonó, Forero & Vidal, Revista Brasil. Bot. 7(1): 68. 1984.

6F214D4E-D16A-5A15-A04F-3D18439BB8C3

Illustration: Forero et al. (1984)

#### Type.

**Brazil. Bahia**: Município de Santa Cruz de Cabrália, 2–4 km a W de Sta. Cruz de Cabrália, pela estrada antiga, área de campos e restinga, 21 Oct 1978 (fr.), *S. A. Mori 10938* (***Holotype***: CEPEC barcode CEPEC 15308!; ***isotypes***: COL!, K!, NY!, RB!).

#### Description.

***Lianas*** or scandent shrubs, ca. 3 m tall; branchlets subglabrous or sparsely pubescent, lenticels abundant, inconspicuous. ***Leaves*** 3–7(–9)-foliolate, congested; petiole 1.1–3 cm long, subglabrous, eglandular; rachis 1.5–3.4 cm long, subglabrous, eglandular; ***leaflets*** subopposite to alternate, pulvinulus ca. 1 mm long; blade of the basal pair of leaflets 1.1–2.8 × 0.5–1.7 cm, ovate, oblong or elliptic, others 1.6–6(–7.2) × 1.3–2.5(–3.7) cm, elliptic or oblong, rarely ovate, chartaceous, discolorous, glabrous on both surfaces, abaxially brownish or greenish, adaxially dull, base slightly asymmetric or symmetric, cordate, subcordate or rounded, apex rounded, rarely narrowly rounded, margin flat or slightly revolute, glabrous; midvein abaxially prominent, adaxially flat or impressed, secondary veins 6–8 pairs, flat or slightly prominent on both surfaces, tertiary veins abaxially flat, adaxially flat or slightly prominent. ***Inflorescences*** in axillary cymes or panicles; bracts 1–2 mm long; peduncle 1.3–2.3 cm long, glabrous or subglabrous, eglandular; rachis 2–4 cm long, glabrous or subglabrous, eglandular; lateral branches 2.4–4.9 cm long, glabrous or subglabrous, eglandular. ***Flowers*** unknown, loosely disposed; buds unknown; pedicel 9–18 mm long, eglandular, 1–2 bracteoles located up to the lower half, deciduous; sepals (persistent on fruits) elliptic, outer surface glabrous or subglabrous, eglandular, inner surface glabrous or subglabrous, margin ciliate, more densely at the apex; petals (Forero et al. 1984) 6–7 × 2 mm, oblong, glabrous on both surfaces; stamens (Forero et al. 1984) connate at base by 1.1–1.2 mm, shorter series 3–4.5 mm long, longer series 5–6 mm long, glabrous; ovary (Forero et al. 1984) 1–1.2 mm long, hirsute, style 2.2–2.5 mm long, sparsely hirsute, stigma bilobate. ***Fruits*** 1.4–1.5 × 0.5–0.6 cm, brownish, outer surface subglabrous, hirsute at the apex, inner surface glabrous, apex obtuse, style partially persistent, calyx covering two thirds of the fruit; ***seeds*** ca. 0.9–1.1 × 0.4–0.6 cm, arillode orangish.

#### Distribution, habitat and phenology.

**Figure 11.**
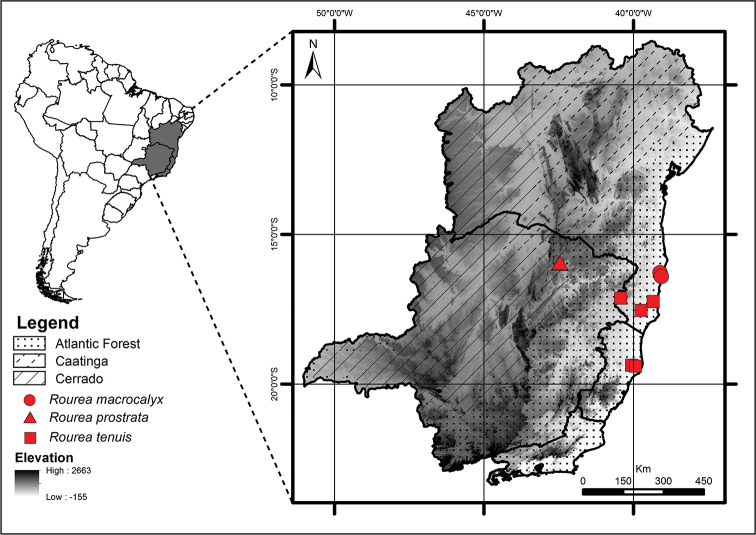
Geographic distribution of *Rourea macrocalyx* (circles), *R. prostrata* (triangles) and *R. tenuis* (squares).

*Rourea macrocalyx* is only known from southern Bahia, occurring in the coastal zone ranging from the municipality of Porto Seguro to Santa Cruz de Cabrália (Fig. 11). This is a lianescent species growing on “Tabuleiro” forests with sandy soils. Specimens have been collected with fruits from October to February.

#### Specimens examined.

**Brazil. Bahia**: Porto Seguro, RPPN VERACEL, ramal para Sta. Cruz Cabrália, ca. 10 km do Centro de visitantes, 05 Feb 2000 (fr.), *J. G. Jardim & M. Alves 2686* (NY); Santa Cruz Cabrália, 16°18'49"S, 39°1'35"W, 11 Oct 2010 (fr.), *B. M. da Silva et al. 24* (HUEFS).

#### Recognition and notes.

*Rourea macrocalyx* is morphologically recognised by the leaves with 3–7(–9) leaflets, pedicel measuring 9–18 mm long and the strongly accrescent calyx, which covers two thirds of the fruits. It is similar to *R. discolor* due to the glabrous leaflets and length of pedicel, but differs mainly by the number of leaflets (3–9 vs. 9–29).

### Rourea martiana

Taxon classificationPlantaeOxalidalesConnaraceae

Baker, in Martius, Fl. Bras. 14(2): 178. 1871.

40C67CEB-19E4-5C78-BAC8-03A4FCCE575C

Fig. 1B

Santalodes martianum (Baker) Kuntze, Revis. Gen. Pl. 1: 155. 1891.

#### Type.

**Brazil. Minas Gerais**: Tabuleiro ad. fl. S. Francisco, prope Salgado, s. d. (fl.), *C. F. P. von Martius 1675* (***Lectotype***: M, first step designated by Schellenberg 1938; ***lectotype***: M barcode M-0243940!, second step designated here; ***isolectotype***: M!).

#### Description.

***Shrubs*** or scandent shrubs, (0.8–)1–3 m tall; branchlets sparsely hirsute to hirsute, lenticels abundant, inconspicuous. ***Leaves*** 9–13(–15)-foliolate, congested or loosely disposed; petiole 0.8–1.8 cm long, sparsely hirsute, with glandular trichomes; rachis 4–6 cm long, sparsely hirsute, with glandular trichomes; ***leaflets*** opposite to subopposite, subsessile; blade of the basal pair of leaflets 0.9–1.8 × 0.6–1.2 cm, orbicular, ovate or narrowly elliptic, others 1.2–3(–4) × 0.7–1.6 cm, oblong, narrowly elliptic or narrowly obovate, chartaceous, slightly discolorous, abaxially hirsute or villous, brownish or greenish, adaxially subglabrous, sparsely hirsute or sparsely villous, dull, base slightly asymmetric or symmetric, cordate, subcordate or rounded, apex rounded, rarely obtuse, margin flat, ciliate; midvein abaxially prominent, adaxially flat, secondary veins ca. 6 pairs, abaxially slightly prominent, adaxially flat, tertiary veins slightly prominent on both surfaces. ***Inflorescences*** in axillary or pseudoterminal cymes; bracts ca. 3 mm long; peduncle 0.9–3.8 cm long, hirsute, with glandular trichomes; rachis 0.5–2.2 cm long, hirsute, with glandular trichomes. ***Flowers*** congested apically; buds 4 × 2.5–3 mm, elliptic or orbicular; pedicel ca. 2 mm long, with glandular trichomes, 2 bracteoles located up to the lower half, persistent; sepals 5 × 2–2.5 mm, chartaceous, ovate, outer surface hirsute, with glandular trichomes, inner surface glabrous or subglabrous, sparsely sericeous at the apex, margin ciliate, more densely at the apex; petals ca. 7 × 2.5 mm, narrowly obovate, glabrous on both surfaces; stamens connate at base by ca. 1 mm, shorter series ca. 5 mm long, longer series ca. 7 mm long, glabrous; ovary ca. 1 mm long, hirsute, style ca. 2 mm long, hirsute, glabrous only at the apex, stigma peltate, bilobate. ***Fruits*** 1–1.2 × 0.4–0.5 cm, orangish or reddish, outer surface subglabrous or sparsely villous, more densely at the apex, inner surface glabrous or subglabrous, apex obtuse, style partially persistent, calyx covering one third of the fruit; ***seeds*** ca. 0.8–0.9 × 0.4–0.5 cm, arillode yellowish.

#### Distribution, habitat and phenology.

This species occurs in central and northeast Minas Gerais and southwest Bahia (Fig. 8). *Rourea martiana* is a shrub, occasionally with climbing branches and grows in the Cerrado or in transitional areas with Caatinga or Atlantic Forest. Specimens have been collected with flowers in April and August and with fruits in October.

#### Specimens examined.

**Brazil. Bahia**: Caetité, 5–8 km S, 21 Oct 1995 (fl.), *G. Hatschbach & J. T. Motta 63237* (RB); Riacho de Santana, Estrada para Igaporã, km 89, 13.7487S, 42.7673W, 12 Oct 2007 (fr.), *J. Paula-Souza et al. 9376* (CTES, SI, SPF). **Minas Gerais**: Grão Mogol, Assentamento Americana. 19 Nov 2014 (fr.), *A. B. Giroldo & J. B. Pereira 330* (CEN); Divisópolis, próximo da cidade, s. d. (fl.), *G. M. Magalhães 15790* (BHCB); Januária, Vale do Peruaçu, Carascal, 26 Oct 1997 (fr.), *A. Salino & L. C. N. Neto 3706* (BHCB, MBM, SPF); Comunidade Boa Vista, 02 Nov 2006 (fr.), *A. C. Sevilha 4597* (CEN); Juramento, Plantar MG 15, Fazenda Tamanduá, 10 Apr 2005 (fl.). *E. Temeirão Neto 4225* (BHCB); *E. Temeirão Neto 4273* (BHCB).

#### Recognition and notes.

*Rourea martiana* is recognised by possessing glandular trichomes, a relatively long-peduncle (0.9–3.8 cm) and a short pedicel (ca 2 mm long).

Baker (1871) described *Rourea martiana* based on the collections of Martius 1675 and Warming 1849, without mentioning the type; the former is only deposited in M, while the latter is deposited in C, GH and K. Schellenberg (1938) inadvertently indicated the lectotype of *R. martiana* (Martius 1675) and described *R. cnestidifolia*, citing Warming 1849 as paratype. Forero (1976) followed Schellenberg’s position, although he called the specimen Martius 1675 holotype of *R. martiana*, and selected Warming 1849 from K as lectotype of *R. cnestidifolia* after the holotype from B (Sellow s. n.) was considered missing. Baker (1871) indeed described *R. martiana* based on two specimens that should be treated as different taxa, so Schellenberg (1938) was right in selecting a type for *R. martiana* and describing *R. cnestidifolia*. The former differs by the middle and apical leaflets up to 4 cm long with usually rounded apex, while in the latter, leaflets are longer than 4 cm with obtuse, acute or narrowly rounded apex.

After fixing the application of *R. martiana* to Martius 1675, however, both Schellenberg (1938) and Forero (1976, 1983) seem to have confused the identity of the species and grouped specimens morphologically distinct and geographically isolated. The type of *R. martiana* was collected in Minas Gerais and, although without a precise location, this specimen matches those collected in central and northeast of the state and south-western Bahia, which are characterised by leaves 9–15-foliolate and a short pedicel (ca. 2 mm long). The specimens, here treated under a new species (*R. diamantina*) – but identified as *R. martiana* by Schellenberg (1938) and Forero (1976, 1983) – are restricted to central Bahia and characterised by leaves 5–9(–13)-foliolate and a longer pedicel (5–13 mm long). Additionally, leaflets in *R. martiana* become slightly larger towards the apex and are usually oblong or narrowly elliptic with rounded apex (Fig. 1B), whereas in *R. diamantina*, they become significantly larger towards the apex and are usually narrowly ovate with obtuse or narrowly-rounded apex (Fig. 7B). Both species are morphologically similar due to the presence of glandular trichomes, leaves and leaflets relatively small and flowers and fruits with similar characteristics. Geographically, these two species are separated by the Chapada Diamantina (Fig. 8), a mountain range located in central Bahia approximately 41,700 km^2^ long and altitudes up to 2,000 m. They also occur in different environments: *R. martiana* grows in areas of Cerrado *s. s.* (occasionally with rocky soils), while *R. diamantina* grows in seasonal forests on Inselbergs.

The position taken by Schellenberg (1938) and Forero (1976, 1983) may be explained because the specimens from Minas Gerais were not available at the time. Nevertheless, the disjunct distribution and the morphological differences are consistent enough to recognise *R. martiana* and *R. diamantina* as distinct species.

The type collection from M is composed of two herbarium sheets mounted separately, but one of them (barcode M-0243940) has an original blue label of Martius, whereas the other (barcode M-0243941) has a different label. According to the *Code* (Turland et al. 2018, Art. 8.3, Ex. 10), these should be regarded as duplicates, so a second step lectotypification is here proposed.

### Rourea prostrata

Taxon classificationPlantaeOxalidalesConnaraceae

C. Toledo, Phytotaxa 408(2): 120. 2019.

26753FCD-272F-596B-9CBA-1D001A8EF677

Illustration: Toledo and Souza (2019)

#### Type.

**Brazil. Minas Gerais**: Rio Pardo de Minas, Vereda Funda, parcela 4, 15°57'20"S, 42°27'23"W, 981 m alt., 11 Dec 2008 (fr.), *A. C. Sevilha et al. 5011* (***Holotype***: CEN barcode CEN 00097193!; ***isotype***: ESA!).

#### Description.

***Subshrubs*** prostrate; branchlets tomentose, lenticels sparse, inconspicuous. ***Leaves*** 13–17(–21)-foliolate, loosely disposed; petiole 0.2–1 cm long, villous, with glandular trichomes; rachis 3–5.5 cm long, villous, with glandular trichomes; ***leaflets*** subopposite, subsessile; blade of the basal pair of leaflets 0.5–1 × 0.3–0.7 cm, orbicular, others 0.9–1.7 × 0.5–1 cm, oblong, rarely elliptic or narrowly obovate, chartaceous, discolorous, abaxially villous, greenish, adaxially sparsely villous, dull, base slightly asymmetric, rounded or subcordate, apex rounded, margin flat, ciliate; midvein abaxially prominent, adaxially flat, secondary veins 5–6 pairs, abaxially slightly prominent, adaxially flat or slightly prominent, tertiary veins flat on both surfaces. ***Inflorescences*** in axillary cymes; bracts ca. 2 mm long; peduncle 0.7–1.8 cm long, hirsute, with glandular trichomes; rachis 1.3–2.2 cm long, hirsute, with glandular trichomes. ***Flowers*** unknown, loosely disposed; pedicel 3–8 mm long, with glandular trichomes, 1–2 bracteoles located up the lower half; sepals (persistent on fruits) with outer surface sparsely hirsute, with glandular trichomes, inner surface subglabrous, margin ciliate; petals (persistent on fruits) glabrous; stamens (persistent on fruits) with sparse glandular trichomes; ovary (persistent on fruits) densely hirsute, style subglabrous, stigma not seen. ***Fruits*** 0.8–1×0.3–0.4 cm, colour not seen, outer surface sparsely hirsute, more densely at the apex, inner surface glabrous, apex acuminate, style partially persistent, calyx covering one third of the fruit; ***seeds*** 0.7–0.8×0.4 cm, arillode colour not seen.

#### Distribution, habitat and phenology.

*Rourea prostrata* in only known from the type location (15°57'20"S, 42°27'23"W), which is near the municipality of Rio Pardo de Minas, MG (Fig. 11). It is the only known species of Connaraceae reported as a prostrate plant. The type was collected with fruits in December.

#### Recognition and notes.

*Rourea prostrata* is morphologically similar to *R. bahiensis* due to the number and size of leaflets. However, *R. prostrata* is a prostrate subshrub with glandular trichomes, while *R. bahiensis* is a liana or scandent shrub eglandular. *Rourea prostrata* can also be mistaken for *R. chrysomalla*, although it mainly differs by its prostrate habit, chartaceous leaflets and sparsely hirsute fruits measuring 0.8–1 × 0.3–0.4 cm vs. erect habit, coriaceous leaflets and completely velutinous fruits measuring 1–1.4 × 0.4–0.6 cm.

### Rourea tenuis

Taxon classificationPlantaeOxalidalesConnaraceae

G. Schellenb., in Engler, Pflanzenreich IV. 127(Heft 103): 199. 1938.

6CC39B82-37FA-548B-9E75-381435B87DA3

**Figure 12.**
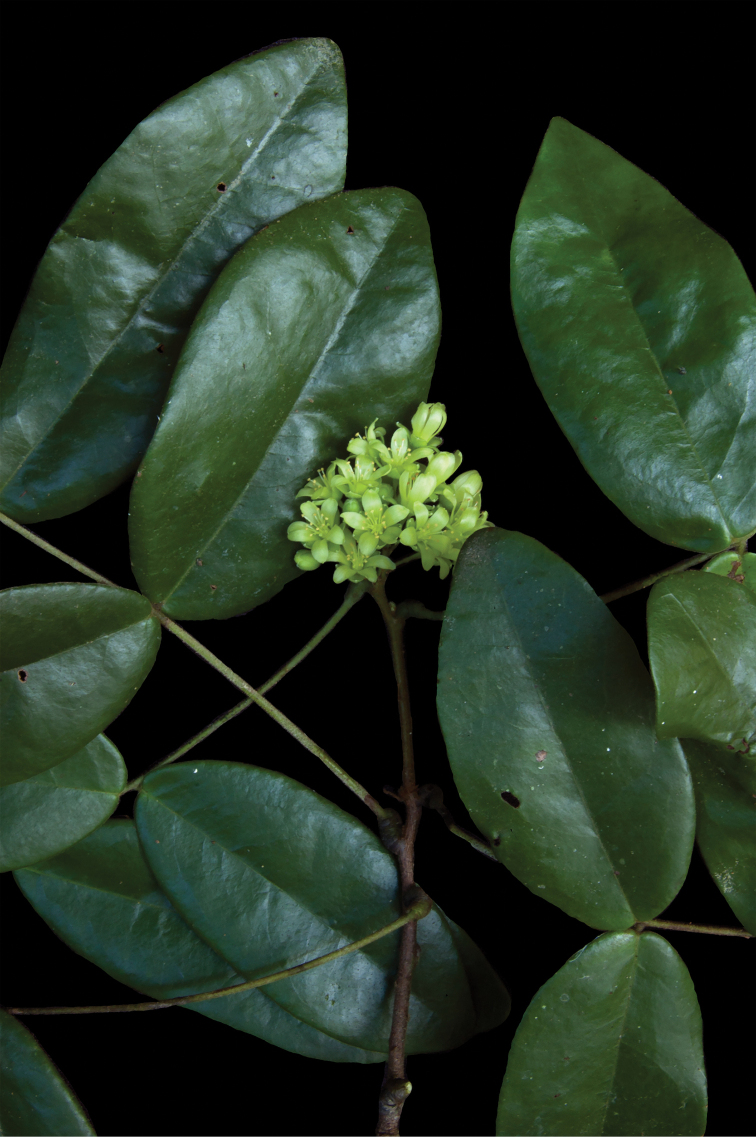
*Rourea tenuis*, flowering branchlet (photo by Thiago Flores).

Figs 1C , 12 ; illustration: Forero et al. (1984

Rourea carvalhoi Forero, Carbonó & L. A. Vidal, Revista Brasil. Bot. 7(1): 72. 1984. Type. Brazil. Bahia: Km. 6 da rod. Teixeira de Freitas a Alcobaça, 9 Oct 1971 (fr.), *T. S. Santos 2094* (*Holotype*: CEPEC 7633!; *isotypes*: COL!, NY!).

#### Type.

**Brazil.** Vittoria, s. d. (fr.), *F. Sellow s. n.* (***Holotype***: B†). **Brazil.** S. d. (fl.), *J. Pohl s. n.* (***Neotype***: BR barcode BR 000000697468!, selected by Forero 1976).

#### Description.

***Lianas*** or scandent shrubs, (0.5–)1–3 m tall; branchlets sparsely hirsute to glabrescent, lenticels absent or sparse, inconspicuous. ***Leaves*** 5–7-foliolate, loosely disposed or congested; petiole 2.7–7 cm long, sparsely hirsute, with glandular trichomes; rachis 2.6–6.5 cm long, sparsely hirsute, with glandular trichomes; ***leaflets*** opposite to subopposite, pulvinulus 1–2 mm long; blade of the basal pair of leaflets 2.3–5.7(–7.7) × 1.4–2.5(–4) cm, elliptic or ovate, others 3.5–8.4(–13) × 2.1–3(–6.7) cm, elliptic or ovate, rarely oblong, chartaceous, slightly discolorous, abaxially sparsely hirsute, more densely on the veins, brownish or greenish, adaxially sparsely hirsute on the veins, dull, base slightly asymmetric, symmetric in the apical leaflet, rounded, obtuse or subcordate, occasionally acute in the apical leaflet, apex obtuse or narrowly rounded, occasionally short-acuminate, margin flat to revolute, ciliate; midvein abaxially prominent, adaxially flat or slightly impressed, secondary veins 5–7 pairs, abaxially slightly prominent, adaxially flat, tertiary veins flat on both surfaces. ***Inflorescences*** in axillary or pseudoterminal determinate thyrses, rarely panicles; bracts ca. 2 mm long; peduncle 1.9–6.4 cm long, hirsute, with glandular trichomes; rachis (0.7–)2–5.8(–7.7) cm long, hirsute, with glandular trichomes; lateral branches 0.5–3 cm long, hirsute, with glandular trichomes. ***Flowers*** loosely disposed or congested apically; buds 2.5–4 × 1.5–2 mm, orbicular or elliptic; pedicel 5–10 mm long, with glandular trichomes, 2 bracteoles located up the lower third, persistent or deciduous; sepals 4–5 × 1–2 mm, chartaceous, ovate, outer surface sparsely hirsute, with glandular trichomes, inner surface glabrous, margin ciliate, more densely at the apex; petals 5–7 × 1.5–2 mm, oblong or narrowly oblong, glabrous or subglabrous on both surfaces; stamens connate at base by ca. 0.8 mm, shorter series 3–3.5 mm long, longer series ca. 5 mm long, glabrous; ovary ca. 1 mm long, densely hirsute, style ca. 1.5 mm long, sparsely villous, subglabrous at the apex, stigma peltate, trilobate to pentalobate. ***Fruits*** 1.3–1.5 × 0.6–0.8 cm, reddish or orangish, outer surface sparsely hirsute, more densely at the apex, inner surface glabrous, apex acuminate, style partially persistent, calyx covering one third of the fruit; ***seeds*** 1.2–1.3 × 0.5–0.7 cm, arillode colour not seen.

#### Distribution, habitat and phenology.

*Rourea tenuis* occurs in Bahia and Espírito Santo, with limits ranging from the municipalities of Itanhém (North) to Linhares (South) (Fig. 11). This species is lianescent and grows in ombrophilous or “Tabuleiro” forests. Specimens have been collected with flowers from April to June and from October to November and with fruits from August to October and in March.

#### Specimens examined.

**Brazil. Bahia**: Porto Seguro, Parque Nacional de Monte Pascoal, área limite entre o PARNA e a Reserva Indígena Barra Velha, da tribo Pataxó, 13 Apr 1998 (fl.), *A. M. Amorim et al. 2530* (CEPEC, NY); Itamarajú, Estrada a Piraji, km 4, 17°15'S, 39°20'W, 1 Jun 1983 (fl.), *R. Callejas et al. 1612* (NY); Itamarajú, 2 km de estrada a Piraji, km 4, 5 Apr 1971 (fl.), *T. S. dos Santos 1558* (NY!); Itanhém, Fazenda Pedra Grande, 16.2 km west of Itanhém on road to Batinga, then 0.6 km north to Fazenda (owner Otevaldo Resende da Silva), 17°07'57.8"S, 40°25'17.8"W, 18 Mar 2001 (fr.), *W. W. Thomas et al. 12351* (CEPEC, NY, RB, SPF). **Espírito Santo**: Reserva Natural Vale, estrada Fruta de arara, 1 Jun 2001 (fl.), *D. A. Folli 3940* (CVRD, ESA); Reserva Natural Vale, estrada Braúna Preta, 12 Apr 2002 (fr.), *D. A. Folli 4352* (CVRD, ESA); Linhares. 5 km S, 5 Aug 1983 (fr.), *G. Hatschbach 46735* (CEPEC, MBM); Floresta Atlântica de Tabuleiro, área com corte seletivo, em regeneração, 19.4000S, 39.9722W, 3 Oct 2000 (fr.), *O. J. Pereira et al. 6541* (VIES).

#### Recognition and notes.

*Rourea tenuis* is recognised by leaves 5–7-foliolate, leaflets usually elliptic or ovate, abaxially hirsute and inflorescences in thyrses or panicles with glandular trichomes.

Forero (1976) included *Rourea tenuis* in Rourea subgen. R. sect. Multifoliolatae mainly due to the long staminal tube, reduced inflorescences and leaflets hairy. Aside from that, this species does not resemble other members of the section in overall similarity of leaves: in *R. tenuis*, the number of leaflets is reduced and they are usually relatively larger, with elliptic or ovate shape (vs. oblong, narrowly elliptic or narrowly ovate) (Fig. 1C). Forero (1976) firstly noticed that *R. tenuis* could be a borderline species between those of R. subgen. R. sect. Multifoliolatae with those from the Amazon, then suggested that the morphological limits of the section should be re-evaluated (Forero et al. 1984). The present study, therefore, considers that the circumscription of the whole group should be tested using molecular and phylogenetic approaches.

## Conclusions and discussion

Rourea subgen. R. sect. Multifoliolatae is a small group of this pantropical genus, with species restricted to south-eastern Brazil and southern Bahia. Most of the species in this section are presumably rare and present restricted distributions, such as *R. blanchetiana*, *R. chrysomalla* and *R. diamantina* and some are only known by the type collection (e.g. *R. barbata* and *R. prostrata*). An exception is *R. glazioui*, which is more widely distributed, very common from north ES to central RJ and well represented in herbarium collections.

Although *Rourea* species have been grouped within different taxonomic ranks (subgenera and sections) over time, none has been tested using molecular data. By comparing the species of R. subgen. R. sect. Multifoliolatae with some *Rourea* from south-eastern Asia, such as *R. fulgens* and *R. mimosoides*, it has been noted that there is a strong morphological resemblance, both in overall similarity of the referred taxa and when particular species are compared (e.g. *R. glazioui* vs. *R. fulgens*). This suggests uncertainty regarding currently-accepted infrageneric classifications. Molecular-based phylogenies are, therefore, essential to achieve a better understanding on the classification, evolution and distribution of the genus.

Similarly, such molecular approaches could also be used to re-evaluate the morphological circumscription traditionally applied to R. subgen. R. sect. Multifoliolatae.

## Supplementary Material

XML Treatment for Rourea Aubl. subgen. Rourea sect. Multifoliolatae

XML Treatment for Rourea bahiensis

XML Treatment for Rourea barbata

XML Treatment for Rourea blanchetiana

XML Treatment for Rourea chrysomalla

XML Treatment for Rourea cnestidifolia

XML Treatment for Rourea diamantina

XML Treatment for Rourea discolor

XML Treatment for Rourea glazioui

XML Treatment for Rourea macrocalyx

XML Treatment for Rourea martiana

XML Treatment for Rourea prostrata

XML Treatment for Rourea tenuis
